# Intracrine FFA4 signaling controls lipolysis at lipid droplets

**DOI:** 10.1038/s41589-025-01982-5

**Published:** 2025-08-05

**Authors:** Shannon L. O’Brien, Emma Tripp, Natasja Barki, Elodie Blondel-Tepaz, Gabrielle Smith, Adam Boufersaoui, Jennie Roberts, Jeremy A. Pike, Joao Correia, Tamara Miljus, Michel Bouvier, Daniel A. Tennant, Brian D. Hudson, Zachary Gerhart-Hines, Graeme Milligan, Thue W. Schwartz, Davide Calebiro

**Affiliations:** 1 Department of Metabolism and Systems Science, College of Medicine and Health, University of Birmingham, Birmingham, UK; 2 Centre of Membrane Proteins and Receptors (COMPARE), Universities of Nottingham and Birmingham, Birmingham, UK; 3 Centre for Translational Pharmacology, School of Molecular Biosciences, College of Medical, Veterinary and Life Sciences, University of Glasgow, Glasgow, UK; 4 Department of Biochemistry and Molecular Medicine, Université de Montréal, Montreal, Quebec Canada; 5 Institute for Research in Immunology and Cancer, Université de Montréal, Montreal, Quebec Canada; 6 Novo Nordisk Foundation Center for Basic Metabolic Research, University of Copenhagen, Copenhagen, Denmark; 7 National Institute for Health and Care Research (NIHR) Birmingham Biomedical Research Centre, Birmingham, UK

**Keywords:** Cell signalling, Lipids, G protein-coupled receptors, Membrane trafficking

## Abstract

G-protein-coupled receptors (GPCRs) can signal from intracellular compartments but the occurrence and relevance of this phenomenon for metabolite-sensing GPCRs is largely unknown. Here, we investigate free fatty acid receptor 4 (FFA4), a metabolite-sensing GPCR activated by medium-chain and long-chain fatty acids. Using live-cell imaging, bioluminescence resonance energy transfer, super-resolution microscopy and cell fractionation, we show that FFA4 localizes to intracellular membranes, particularly endoplasmic reticulum subdomains surrounding lipid droplets, in both immortalized adipocytes and mouse adipose tissue. Upon lipolysis, locally released fatty acids appear to rapidly activate this intracellular FFA4 pool, leading to G_i__/o_ protein signaling and preferential reduction of cyclic adenosine monophosphate levels near lipid droplets. These mechanisms are required for efficient FFA4-mediated lipolysis regulation, as shown using tethered mini-Gα_i/o_ proteins to locally inhibit G_i/o_ signaling. These findings reveal an unexpected ‘intracrine’ GPCR signaling modality involved in the local regulation of cell metabolism, with biological and pharmacological implications.

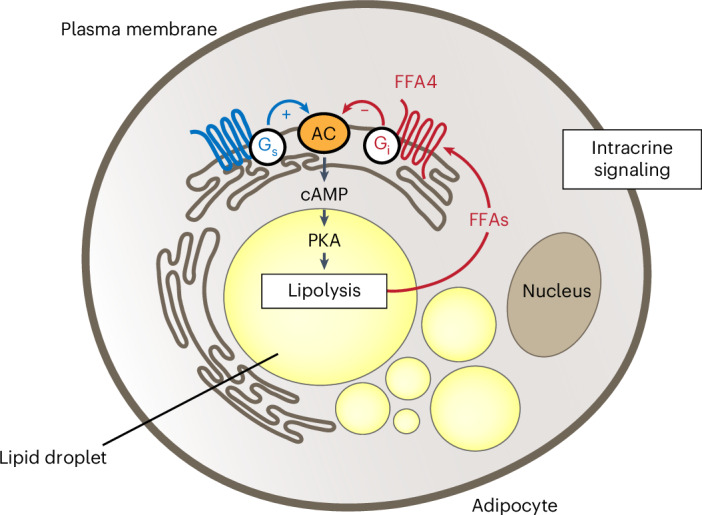

## Abstract (web summary)

An ‘intracrine’ signaling mechanism is proposed whereby a G-protein-coupled receptor (free fatty acid receptor 4) senses locally released fatty acids on intracellular membranes associated with lipid droplets to efficiently regulate lipolysis in adipocytes.

## Main

Metabolites are essential energy sources and building blocks for biosynthetic pathways^1^. Traditionally, they were mainly thought to modulate metabolic pathways by directly influencing the activity of key biosynthetic enzymes. However, with the deorphanization of several GPCRs, it has become apparent that metabolites can also modulate cellular functions by activating a growing number of metabolite-sensing GPCRs^1^.

In parallel, although originally believed to be active only at the plasma membrane (PM), evidence accumulated over the past 15 years has indicated that GPCRs can also signal through G proteins from intracellular sites, such as early endosomes or the Golgi and *trans*-Golgi network, to regulate cellular functions locally^2–6^. Given the emerging paradigm of GPCR signaling at intracellular sites and the compartmentalized nature of intracellular metabolism^7^, an intriguing hypothesis is that metabolite-sensing GPCRs might signal from intracellular membranes to locally regulate metabolic pathways. However, the potential occurrence and physiological relevance of metabolite-sensing GPCR signaling at intracellular sites remains to be clarified.

A prime example of a tightly regulated metabolic pathway is lipolysis, stimulated by hormones like adrenaline acting upon G_s_-coupled receptors in adipocytes. The resulting increase in intracellular cAMP levels and protein kinase A activation trigger the release of free fatty acids (FFAs) that are stored as triglycerides in lipid droplets (LDs). FFAs, in turn, have been shown to activate FFA4 (also known as GPR120), a GPCR that responds to medium-chain and long-chain FFAs^8^ and is highly expressed in adipocytes^1,9^. Recent studies suggest that FFA4 might have an important role in regulating adipocyte metabolism through a negative feedback loop that inhibits lipolysis^10,11^. However, the underlying molecular mechanisms are insufficiently understood.

Here, we use real-time bioluminescence resonance energy transfer (BRET) measurements, live-cell imaging, mice expressing endogenous hemagglutinin (HA)-tagged FFA receptors and functional readouts to investigate the spatiotemporal organization of FFA4 signaling within adipocytes. Surprisingly, we find that FFA4 predominantly resides and signals on intracellular membranes associated with LDs, which appears to be required for FFA4 to efficiently modulate lipolysis in an ‘intracrine’ manner.

## Results

### FFA4 signals through G_i/o_ on HEK293 intracellular membranes

**Extended Data Fig. 1:**
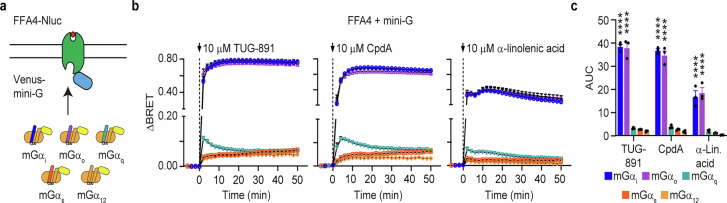
FFA4 is primarily coupled to Gα_i/o_ in HEK293T cells. **a**, Schematic of the BRET assay used to measure FFA4 coupling specificity with mini-G probes. **b**, Agonist-dependent increases in Venus-tagged mini-G probe recruitment to FFA4-Nluc upon stimulation with TUG-891, Compound A (CpdA) or α-linolenic acid (α-Lin. acid), monitored by real-time BRET measurements. **c**, Corresponding AUC values. Data are mean ± SEM of n = 3 independent biological replicates. Results in **c** are statistically significant by two-way ANOVA with Dunnett’s post-hoc test. ****p < 0.0001 vs corresponding mGα_q_ response. Source data

**Extended Data Fig. 2:**
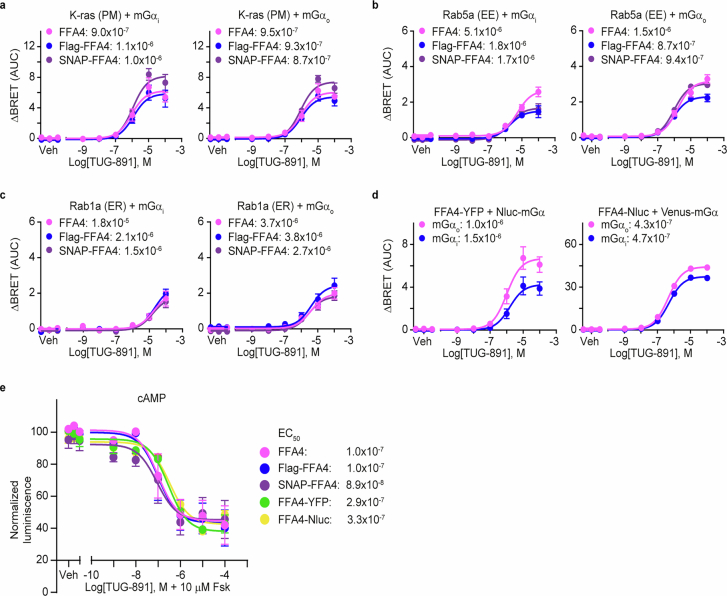
Functional validation of the employed tagged FFA4 constructs. HEK293T cells were transfected with the indicated constructs and stimulated with increasing concentrations of TUG-891. **a-c**, Activation of tagged FFA4 constructs. FFA4 activation was monitored by BRET between Nluc-tagged mGα_i_ (left) or mGα_o_ (right) and Venus-tagged K-ras (PM) (**a**), Rab5a (early endosomes, EE) (**b**) or Rab1a (ER) (**c**). Wild-type FFA4 was included for comparison. **d**, Activation of YFP- and Nluc-tagged FFA4 constructs. FFA4 activation was monitored by BRET between FFA4-YFP and Nluc-tagged mGα_i/o_ (left) or FFA4-Nluc and Venus-tagged mGα_i/o_ (right). **e**, Ability of tagged FFA4 constructs to inhibit cAMP production. HEK293T cells were co-transfected with the indicated constructs together with the cAMP GloSensor. Cells were stimulated with forskolin alongside increasing concentrations of TUG-891. Wild-type FFA4 was included for comparison. Listed are the estimated EC_50_ values for each construct. Data are mean ± SEM of n = 5 (Flag-FFA4, SNAP-FFA4), 3 (FFA4) (**a**), 7 (Flag-FFA4, SNAP-FFA4), 4 (FFA4) (**b**), 3 (**c**), 4 (FFA4-YFP), 3 (FFA4-Nluc) (**d**) and 4 (**e**) independent biological replicates. Source data

Whereas FFA4 was originally thought to be primarily coupled to G_q__/11_ (refs. ^12,13^), recent studies in undifferentiated cells have additionally revealed G_i/o_ coupling^11,14,15^, which could directly explain its inhibitory effects on lipolysis. To further investigate this, we performed real-time BRET measurements between FFA4 carrying NanoLuc luciferase (Nluc) at its C terminus (FFA4–Nluc) and Venus-tagged mini-G probes (mGα_i_, mGα_o_, mGα_q_, mGα_s_ or mGα_12_), which consist of engineered guanosine triphosphatase (GTPase) domains of Gα subunits that retain coupling specificity and rapidly translocate from the cytoplasm to active GPCRs^16^ (Extended Data Fig. 1a). Stimulation with two selective synthetic FFA4 agonists, TUG-891 (ref. ^13^) and compound A (CpdA)^17^, as well as the endogenous agonist α-linolenic acid^8^, caused robust mGα_i/o_ and only minor mGα_q/s/12_ translocation in transfected HEK293T cells (Extended Data Fig. 1b,c), confirming strong coupling to G_i/o_. All tagged FFA4 constructs used in the study were functional, with half-maximal effective concentration values for mini-G recruitment and cAMP inhibition comparable to wild-type FFA4 (Extended Data Fig. 2).

**Extended Data Fig. 3:**
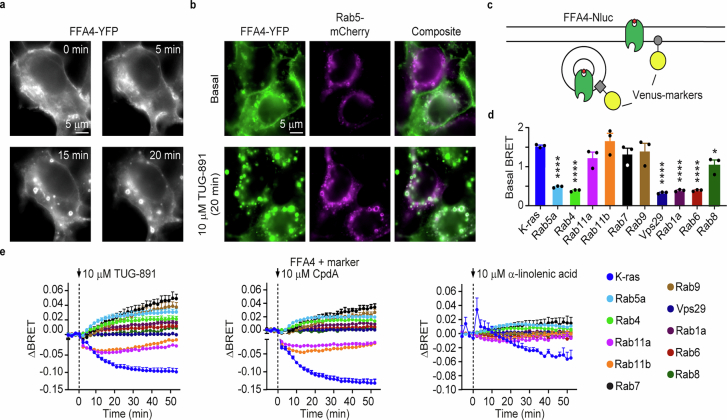
FFA4 traffics to intracellular compartments in HEK293T cells. **a**, Effect of TUG-891 stimulation on FFA4 localization. Shown are selected frames from a representative HILO image sequence of HEK293T cells transfected with FFA4-YFP. **b**, Colocalization of FFA4-YFP with an early endosome marker (Rab5-mCherry) in HEK293T cells stimulated with TUG-891. White color indicates colocalization. **c**, Schematic illustrating the bystander BRET assay used to monitor FFA4 internalization and intracellular trafficking in real-time. **d**, Results of basal bystander BRET measurements between FFA4-Nluc and Venus-tagged subcellular markers, that is K-ras (PM), Rab5a (early endosomes), Rab4 (fast recycling endosomes), Rab11a/b (slow recycling endosomes), Rab7 (late endosomes/lysosomes), Rab9 (late endosomes to trans-Golgi network), Vps29 (retrograde trafficking from early endosome to trans-Golgi network), Rab1a (ER to the *cis*-Golgi), Rab6 (Golgi and trans-Golgi network), and Rab8 (trans-Golgi network to the PM). **e**, Agonist-dependent changes in FFA4 localization at subcellular compartments, monitored by real-time bystander BRET between FFA4-Nluc and Venus-tagged subcellular markers. Data are mean ± SEM of n = 3 independent biological replicates. Images are representative of 8 (**a**) and 3 (**b**) independent experiments. Results in **d** are statistically significant by one-way ANOVA followed by Dunnett’s post-hoc test. ****p < 0.0001, *p < 0.05 vs K-ras. Source data

We next investigated FFA4 trafficking. As a first approach, we performed highly inclined thin illumination (HILO) microscopy experiments in HEK293T cells transfected with yellow fluorescent protein (YFP)-tagged FFA4 (FFA4–YFP). This technique enables high-speed, sensitive visualization of dynamic trafficking and signaling events in living cells^5^. Under basal conditions, FFA4 was localized predominantly at the PM, with a smaller fraction at intracellular compartments (Extended Data Fig. 3a). Upon stimulation with TUG-891, FFA4 rapidly internalized to early endosomes, visualized by Rab5–mCherry cotransfection (Extended Data Fig. 3b), and possibly other compartments. Moreover, we used a bystander BRET assay that monitors the proximity between FFA4–Nluc and Venus-tagged subcellular markers in real time^18^ (Extended Data Fig. 3c). Basal measurements showed FFA4 at the PM (K-ras), recycling endosomes (Rab11a and Rab11b), late endosomes (Rab7 and Rab9) and the *trans*-Golgi network (Rab8 and Rab9) (Extended Data Fig. 3d). Upon TUG-891 stimulation, FFA4 rapidly redistributed from the PM and recycling endosomes to early endosomes (Rab5a), late endosomes (Rab7 and Rab9), the *trans*-Golgi network (Rab9 and Rab6), fast recycling endosomes (Rab4) and the endoplasmic reticulum (ER; Rab1a); no relevant changes were observed with retromer (Vps29) or *trans*-Golgi network to PM (Rab8) markers (Extended Data Fig. 3e). Comparable trafficking profiles were observed with CpdA and α-linolenic acid, despite α-linolenic acid being less effective at the tested (10 μM) concentration (Extended Data Fig. 3e).

**Extended Data Fig. 4:**
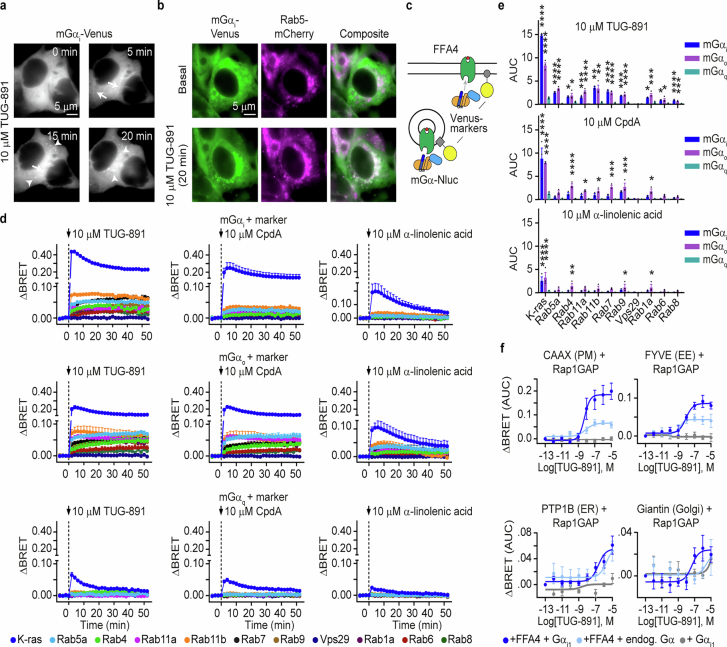
FFA4 signals from intracellular compartments in HEK293 cells. **a**, Effect of TUG-891 stimulation on Venus-mGα_i_ translocation. Shown are selected frames from a representative HILO image sequence of cells co-transfected with FFA4 and Venus-mGα_i_. Arrows, PM. Arrowheads, endosomes. **b**, Colocalization of Venus-mGα_i_ with an early endosome marker (Rab5-mCherry). **c**, Schematic illustrating the bystander BRET assay used to monitor mGα_i_ translocation to subcellular compartments in real-time. **d**, Agonist-dependent translocation of Nluc-mGα_i_ (top), Nluc-mGα_o_ (middle) or Nluc-mGα_q_ (bottom) to Venus-tagged subcellular markers, that is K-ras (PM), Rab5a (early endosomes), Rab4 (fast recycling endosomes), Rab11a/b (slow recycling endosomes), Rab7 (late endosomes/lysosomes), Rab9 (late endosomes to trans-Golgi network), Vps29 (retrograde trafficking from early endosome to trans-Golgi network), Rab1a (ER to the *cis*-Golgi), Rab6 (Golgi and trans-Golgi network), and Rab8 (trans-Golgi network to the PM) in cells co-transfected with wild-type FFA4. **e**, Corresponding AUC values. **f**, Agonist dependent G_i/o_ protein activation at subcellular compartments monitored by ebBRET between RlucII-tagged Rap1GAP and rGFP fused to CAAX (PM), FYVE (early endosomes, EE), PTP1B (ER) or Giantin (Golgi apparatus). Cells either expressed endogenous G proteins or were co-transfected with Gα_i1_, as indicated. Data are mean ± SEM of n = 4 (Venus-mGα_i_ upon TUG-891 stimulation), 3 (Venus-mGα_o_ upon TUG-891 stimulation, CpdA and α-linolenic acid stimulation) (**d**, **e**) and 5 (CAAX), 4 (FYVE, PTP1B) (**f**) independent biological replicates. Images are representative of 5 (**a**), and 3 (**b**) independent experiments. Results in **e** are statistically significant by one-way ANOVA followed by Dunnett’s post-hoc test. ****p < 0.0001, ***p < 0.001, **p < 0.01, *p < 0.05 vs corresponding mGα_q_ response. Source data

To investigate the spatiotemporal pattern of FFA4 activation, we transfected HEK293T cells with FFA4 and Venus-tagged mini-G probes and imaged them by HILO microscopy. TUG-891 stimulation caused a rapid recruitment of mGα_i_ to the PM (Extended Data Fig. 4a), followed by early endosomes (Extended Data Fig. 4a,b) and other intracellular membranes. We then performed bystander BRET measurements between mini-G probes fused to Nluc and Venus-tagged subcellular markers (Extended Data Fig. 4c). TUG-891, CpdA and α-linolenic acid stimulation induced rapid and strong mGα_i/o_ recruitment to the PM (K-ras) and, to a variable extent, all investigated intracellular compartments except the retromer compartment (Vps29) (Extended Data Fig. 4d,e). By contrast, only modest recruitment of mGα_q_ was observed to the PM, with negligible recruitment to intracellular compartments (Extended Data Fig. 4d,e). In addition, we used an orthogonal enhanced bystander BRET (ebBRET) assay for G_i/o_ protein activation^15,19,20^. This assay monitors effector membrane translocation as BRET between the RlucII-tagged Gα_i/o_-binding domain of Rap1 GTPase-activating protein (Rap1GAP) and an independent set of *Renilla* green fluorescent protein (rGFP)-tagged subcellular markers^15,19,20^. TUG-891 stimulation induced concentration-dependent Rap1GAP recruitment to the PM (CAAX), in addition to early endosomes (FYVE), the ER (PTP1B) and, to a lesser extent, the Golgi apparatus (Giantin) (Extended Data Fig. 4f).

Altogether, these results suggest that, in HEK293 cells, FFA4 localizes primarily to the PM and, upon agonist stimulation, internalizes and signals through G_i/o_ from the PM and possibly various intracellular compartments.

### FFA4 signals on LD-associated membranes in adipocytes

**Fig. 1:**
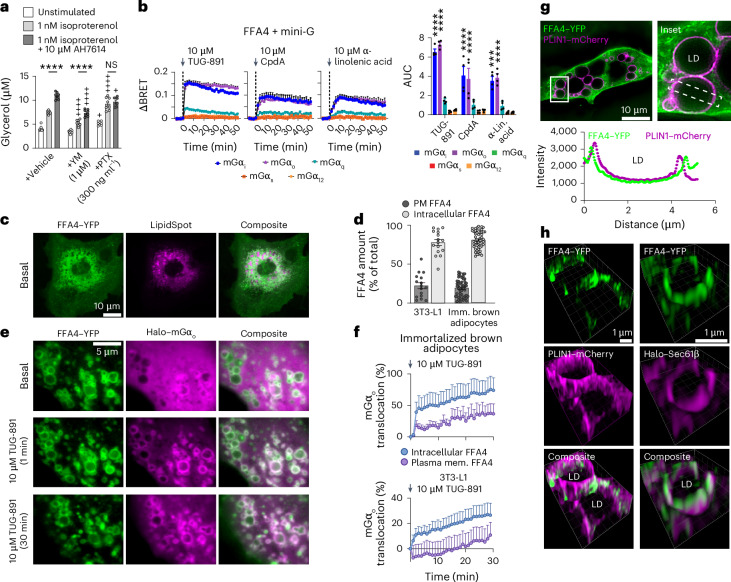
FFA4 resides at and signals from an intracellular compartment intimately associated with LDs in adipocytes. **a**, Evidence for G_i/o_ signaling by endogenous FFA4 in differentiated immortalized brown adipocytes. Cells were pretreated with a G_q/11_ inhibitor (YM-254890, YM), G_i/o_ inhibitor (pertussis toxin, PTX) or vehicle, followed by isoproterenol to induce lipolysis, in the presence or absence of a partial FFA4 inhibitor (AH7614). Lipolysis was assessed as glycerol released into the cell culture medium. **b**, Recruitment of Venus-tagged mini-G probes to FFA4–Nluc upon TUG-891, CpdA or α-linolenic acid stimulation, monitored by real-time BRET in differentiated immortalized brown adipocytes. Left, BRET traces. Right, corresponding area under the curve (AUC) values. **c**, HILO images of FFA4–YFP with an LD stain (LipidSpot) in differentiated 3T3-L1 cells. **d**, Percentage of FFA4–YFP localized at the PM versus intracellular membranes. **e**, Representative HILO image sequence of Halo–mGα_o_ recruitment to FFA4–YFP upon TUG-891 stimulation in differentiated 3T3-L1 cells. **f**, Quantification of mGα_o_ translocation to FFA4 at the PM or intracellular compartments upon TUG-891 stimulation. **g**, SIM images of differentiated immortalized brown adipocytes cotransfected with FFA4–YFP and an LD membrane marker (PLIN1–mCherry). Top, representative image and zoomed-in inset showing close association of FFA4 with LD membranes. Bottom, intensity profiles across the dashed box. **h**, Zoomed-in 3D SIM images of differentiated immortalized brown adipocytes cotransfected with FFA4–YFP and PLIN1–mCherry (left) or an ER marker (Halo–Sec61β) (right). Data are the mean ± s.e.m. of *n* = 8 (**a**) and 4 (**b**) independent biological replicates, *n* = 16 and 53 cells (**d**) and *n* = 17 and 13 cells (**f**). Images are representative of *n* = 4 (**c**), 5 (**e**), 3 (**g**) and 3 (**h**) independent experiments. Results were determined to be statistically significant according to a two-way ANOVA followed by Šidák’s multiple-comparison test (**a**) or Dunnett’s post hoc test (**b**). ****P < 0.0001 (**a**); ++++*P* < 0.0001 and +*P* < 0.05 versus corresponding vehicle (**a**); ****P < 0.0001 and ***P < 0.001 versus corresponding mGα_q_ response (**b**); NS, statistically not significant. Source data

We then investigated FFA4 localization and signaling in adipocytes, using the 3T3-L1 preadipocyte cell line^21^ and mouse immortalized brown adipocytes^22^, which we differentiated in vitro. Pretreatment of differentiated immortalized brown adipocytes with an FFA4 negative allosteric modulator (AH7614; that is, a partial FFA4 inhibitor) enhanced lipolysis induced by the β-adrenergic agonist isoproterenol, confirming the expression of functional endogenous FFA4 (Fig. 1a). The AH7614 effect was G_i/o_-dependent, as it was prevented by pertussis toxin; for comparison, the G_q/11_ inhibitor YM-254890 reduced all responses, consistent with a positive effect of G_q/11_ on lipolysis, but did not block the AH7614 effect (Fig. 1a). The higher transfection efficiency in immortalized brown adipocytes also allowed us to investigate FFA4 coupling specificity by BRET, which revealed a predominant G_i/o_ coupling as in HEK293T cells (Fig. 1b).

**Extended Data Fig. 5:**
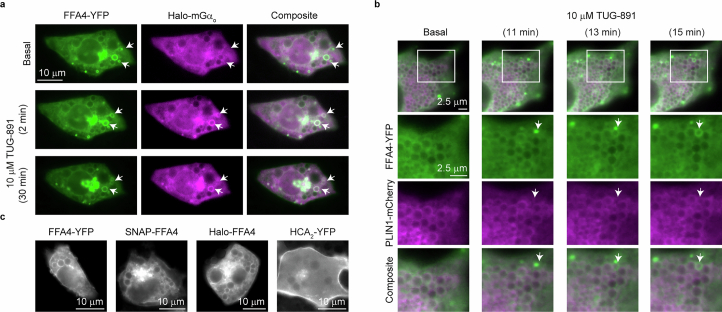
FFA4 localization and signaling in differentiated immortalized brown adipocytes. **a**, Representative HILO image sequences of Halo-mGα_o_ recruitment to FFA4-YFP (arrows) upon stimulation with TUG-891 in differentiated immortalized brown adipocytes. **b**, Representative HILO image sequence showing a vesicle carrying FFA4-YFP apparently fusing with membranes belonging to the LD-associated compartment (arrow) in a differentiated immortalized brown adipocyte stimulated with TUG-891. **c**, HILO images of differentiated immortalized brown adipocytes transfected with FFA4-YFP, SNAP-FFA4, Halo-FFA4 or HCA2-YFP. Images are representative of 5 (**a**), 3 (**b**), and 2 (**c**) independent experiments.

We next probed the subcellular localization of transiently transfected YFP-tagged FFA4, which we quantified by training a machine-learning-based pixel classifier in Ilastik^23^ (Methods). Remarkably, in both differentiated 3T3-L1 and immortalized brown adipocytes, a large proportion (77.7% ± 3.3% and 80.8% ± 1.5%, respectively) of transfected FFA4 was located on intracellular membranes under basal conditions, with only a smaller fraction at the PM (Fig. 1c,d and Extended Data Fig. 5a). The intracellular compartment containing FFA4 appeared to be intimately associated with LDs, labeled with a fluorescent lipid stain (LipidSpot) (Fig. 1c). Upon stimulation with TUG-891, FFA4 located at the PM internalized to small endosomal vesicles that slowly accumulated near LDs. Occasionally, vesicles carrying FFA4 appeared to later fuse with the preexisting LD-associated membrane compartment (Extended Data Fig. 5b). Similar localization patterns were observed using FFA4 N-terminally tagged with SNAP or Halo (Extended Data Fig. 5c) but not with another YFP-tagged metabolite GPCR, the hydroxycarboxylic acid receptor 2 (HCA2), which was predominantly at the PM (Extended Data Fig. 5c), suggesting that FFA4 association with LDs was specific and not just a result of the tags used.

We next monitored mGα_o_ translocation using HILO imaging (Fig. 1e and Extended Data Fig. 5a), followed by Ilastik classification and quantifications in MATLAB to compare FFA4 activation at the PM and intracellular membranes (Fig. 1f). Remarkably, stimulation with the cell-permeable FFA4 agonist TUG-891 caused translocation of mGα_o_ to the intracellular pool of FFA4 intimately associated with LDs, with smaller translocation to the PM FFA4 pool. The intracellular response was particularly rapid, occurring already approximately 1 min after stimulation, a time that preceded FFA4 internalization and appearance on new endosomal vesicles.

To further investigate the subcellular localization of FFA4 in adipocytes, we resorted to super-resolution structured illumination microscopy (SIM). Super-resolved three-dimensional (3D) images obtained in differentiated immortalized brown adipocytes cotransfected with FFA4–YFP and mCherry-tagged perilipin 1 (PLIN1), used as an LD membrane marker, confirmed that FFA4–YFP was located in close proximity to the surface of LDs (Fig. 1g,h) (Pearson’s coefficient: 0.42 ± 0.19). Because the LD membrane consists of a single phospholipid monolayer that is unlikely to accommodate integral membrane proteins such as GPCRs, we reasoned that FFA4 should rather be localized on membranes of another, closely associated membrane compartment. Given the known intimate association of the ER with LDs^24,25^ and the pattern observed in the SIM images, the ER appeared as a likely candidate. We, therefore, conducted additional SIM experiments in cells cotransfected with FFA4–YFP and an ER marker (Halo–Sec61β) (Fig. 1h). The results showed a distinct colocalization of FFA4–YFP with Sec61β-positive ER subdomains (Pearson’s coefficient: 0.78 ± 0.08).

Altogether, these results indicate that, in immortalized adipocytes, a relevant fraction of overexpressed FFA4 resides in close proximity to LDs before stimulation, likely on membrane subdomains of the ER, where it can be rapidly activated by cell-permeable agonists.

### Endogenous FFA4 is located intracellularly in adipose tissue

**Fig. 2:**
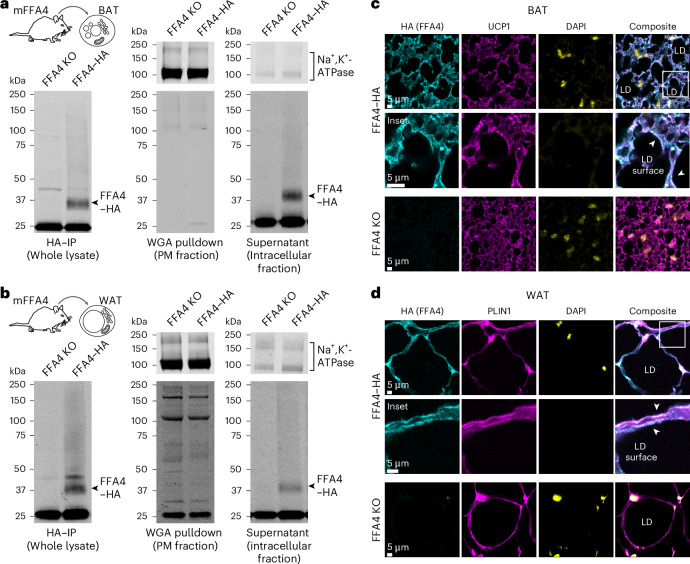
Endogenous FFA4 is present on intracellular membranes in mouse adipose tissue. **a**,**b**, Results of cell fractionation experiments in BAT (**a**) and WAT (**b**) lysates obtained from transgenic knock-in mice expressing endogenous FFA4–HA. Lysates were pulled down with WGA beads to separate the PM fraction from the intracellular fraction. Lysates were probed with anti-HA antibody to detect HA-tagged receptors. Na^+^,K^+^-ATPase was used as a PM marker. **c**,**d**, FFA4 immunofluorescence in BAT (**c**) and WAT (**d**) slices from FFA4–HA mice. FFA4 was detected with an anti-HA antibody (cyan). The slices were costained with the BAT marker UCP1 (**c**) or PLIN1 (**d**) (magenta). Nuclei were stained with DAPI (yellow). FFA4-knockout (FFA4 KO) mice were used as a negative control. Western blots are representative of *n* = 3 (**a**) and 4 (**b**) independent experiments. Immunofluorescence images are representative of *n* = 3 independent experiments. Source data

**Extended Data Fig. 6:**
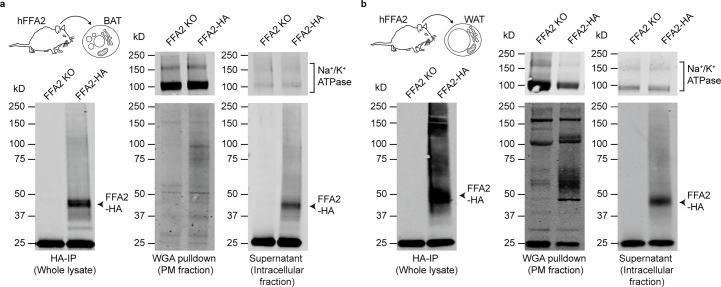
Subcellular localization of endogenous FFA2 in mouse adipose tissue. **a**, **b**, Results of cell-fractionation experiments in BAT (**a**) and WAT (**b**) tissue lysates obtained from transgenic knock-in mice expressing human FFA2 with a C-terminal HA tag under the endogenous FFA2 promoter (FFA2-HA). Lysates were pulled down with wheat germ agglutinin (WGA) beads to separate the plasma membrane (PM) fraction from the intracellular fraction. Lysates were probed with an anti-HA antibody to detect HA-tagged receptors. Na^+^/K^+^ ATPase was used as PM marker. CRE-MINUS mice that harbor hFFA2-HA at the mouse Ffar2 locus but whose expression has not been induced (FFA2 KO) were used as negative control. Western blots are representative of 3 (BAT), 4 (WAT) independent experiments. Source data

To investigate the subcellular localization of endogenous FFA4 in native tissue, we took advantage of knock-in mice expressing endogenous FFA4 with an HA tag fused to its C terminus (FFA4–HA)^26^. We first performed cell fractionation experiments in brown adipose tissue (BAT) and white adipose tissue (WAT) lysates, using wheat germ agglutinin (WGA) to separate the PM from intracellular organelles. In agreement with our observations in 3T3-L1 cells and immortalized brown adipocytes, a major fraction of FFA4 was found intracellularly and we were in fact unable to detect FFA4 at the cell surface in both BAT (Fig. 2a) and WAT (Fig. 2b). No specific signal was detected in lysates from FFA4-knockout mice, used as a negative control (Fig. 2a,b). For comparison, endogenous FFA2 could be detected both at the PM and intracellular sites in WAT tissue of FFA2–HA knock-in mice^27^ (Extended Data Fig. 6). In addition, we performed immunofluorescence on BAT (Fig. 2c) and WAT (Fig. 2d) slices obtained from the FFA4–HA mice. In agreement with the cell fractionation results, a major fraction of FFA4 was observed intracellularly and surrounding LDs (Fig. 2c,d), showing colocalization with the LD membrane marker PLIN1 costained in WAT (Pearson’s coefficient: 0.76 ± 0.04).

### Evidence for intracrine FFA4 signaling in adipocytes

On the basis of our results, we hypothesized that the intracellular FFA4 pool might be activated by FFAs released during lipolysis, which we investigated with additional experiments.

**Fig. 3:**
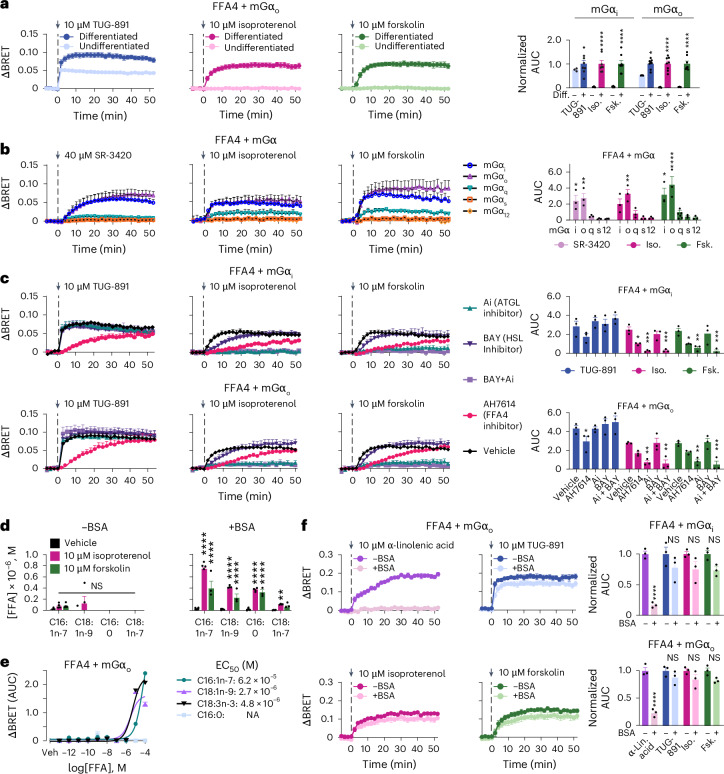
Endogenous FFAs released during lipolysis activate FFA4 in an intracrine manner. **a**, Real-time BRET monitoring of mGα_i/o_ recruitment to FFA4 in response to TUG-891, isoproterenol or forskolin stimulation in undifferentiated versus differentiated immortalized brown adipocytes. Cells were transfected with Venus-tagged mGα_i/o_ and FFA4–Nluc. Left, BRET traces. Right, corresponding normalized AUC values. **b**, Comparison of mGα_i/o/q/s/12_ recruitment to FFA4 in differentiated immortalized brown adipocytes. Left, BRET traces. Right, corresponding AUC values. **c**, Effects of pretreatment (20 min) with an ATGL inhibitor (atglistatin (Ai); 10 μM), HSL inhibitor (BAY, 5 μM) or FFA4 negative allosteric modulator (AH7614; 10 μM) on mGα_i/o_ recruitment to FFA4. Left, BRET traces. Right, corresponding AUC values. **d**, Extracellular fatty acid concentrations detected by GC–MS after 60-min stimulation with isoproterenol, forskolin or vehicle in the absence (−BSA) or presence (+BSA) of 100 μM fatty-acid-free BSA. **e**, mGα_o_ recruitment to FFA4 in response to increasing concentrations of C16:1n-7 (palmitoleic acid), C18:1n-9 (oleic acid), C18:3n-3 (α-linolenic acid) or C16:0 (palmitic acid). **f**, Effect of fatty-acid-free BSA (10 μM) on mGα_i/o_ recruitment to FFA4. Left, BRET traces. Right, corresponding AUC values. Mini-G translocation in **b**,**c**,**f** was measured by BRET as in **a**. Data are the mean ± s.e.m. of *n* = 3 (**a**, undifferentiated adipocytes), 8 (**a**, differentiated adipocytes), 3 (**b**,**c**,**e**,**f**) and 4 (**d**) independent biological replicates. Results were determined to be statistically significant according to a two-way ANOVA followed by Šidák’s multiple-comparison test (**a**) or Dunnett’s post hoc test (**b**–**d**,**f**). ****P < 0.0001, ***P < 0.001, **P < 0.01 and *P < 0.05 versus corresponding undifferentiated condition (**a**), mGα_q_ (**b**), corresponding vehicle (**c**,**d**) or corresponding −BSA condition (**f**). Source data

In undifferentiated immortalized brown adipocytes, which only contain a few LDs and have negligible lipolytic activity, direct stimulation with TUG-891 caused robust FFA4 activation, measured as mGα_i/o_ recruitment to FFA4 by BRET, whereas no detectable response was observed upon lipolysis induction with forskolin or isoproterenol (Fig. 3a). By contrast, in differentiated immortalized brown adipocytes, both forskolin and isoproterenol stimulation induced strong and rapid FFA4 activation (Fig. 3a). Furthermore, we took advantage of SR-3420 (ref. ^28^), which activates lipolysis independently of cAMP and protein kinase A by binding to CGI-58. This causes the release of CGI-58 from PLIN and subsequent activation of adipose triglyceride lipase (ATGL), the enzyme that catalyzes the first and rate-limiting step of triglyceride hydrolysis^29^. Direct lipolysis stimulation with SR-3420 induced mGα_i/o_ recruitment to FFA4 similar to isoproterenol and forskolin; for comparison, only minor mGα_q_ and no detectable mGα_s/12_ recruitment were observed (Fig. 3b).

To further investigate our hypothesis, we took advantage of two lipolysis inhibitors, that is, the ATGL inhibitor atglistatin and BAY 59-9435 (BAY), targeting the hormone-sensitive lipase (HSL), which catalyzes the second key step of triglyceride hydrolysis^29^. Atglistatin virtually blocked the activation of FFA4 induced by isoproterenol or forskolin (Fig. 3c), whereas BAY delayed the response but did not affect its amplitude (Fig. 3c). Importantly, neither treatment alone or in combination affected the direct activation of FFA4 with TUG-891, used as a control (Fig. 3c). Furthermore, all responses were similarly delayed and reduced by the addition of the FFA4 negative allosteric modulator AH7614 (Fig. 3c).

We next asked whether the rapid activation of FFA4 by endogenously released FFAs occurred through an intracrine as opposed to an autocrine or paracrine mechanism. To begin with, we measured FFAs released into the cell culture medium by a sensitive gas chromatography–mass spectrometry (GC–MS) method (detection limit of ~400 pM). Under the experimental conditions used for the above BRET experiments, FFA concentrations in the culture medium remained indistinguishable from a blank control sample even after stimulation with 10 μM forskolin or isoproterenol for 60 min (Fig. 3d). Detectable increases in palmitoleic (C16:1n-7), palmitic (C16:0), oleic (C18:1n-9) and vaccenic (C18:1n-7) acid concentrations were only present upon the addition of BSA to the culture medium, which facilitates FFA export and medium accumulation^30^ (Fig. 3d). We then measured mGα_o_ recruitment to FFA4 in response to increasing concentrations of palmitoleic, palmitic, oleic and α-linolenic acid, which are reported FFA4 agonists^12,31,32^. Under the conditions used for our signaling experiments, which were conducted in the absence of BSA, detectable FFA4 activation was only observed for FFA concentrations in the micromolar range (Fig. 3e). This is much higher than the upper boundary of our estimate for the FFAs in the culture medium after forskolin or isoproterenol stimulation, suggesting that the concentrations of FFAs released in the culture medium are most likely too low to activate cell surface FFA4. Of note, although palmitic acid was previously reported to be a FFA4 agonist in some^33^ but not all studies^12^, no response was detected for palmitic acid concentrations up to 100 μM, suggesting that it is not a physiologically relevant FFA4 agonist.

To further test our hypothesis, we reasoned that, as we previously demonstrated studying FFA1 (also known as GPR40)^34^, adding an excess of BSA to the culture medium should buffer extracellular fatty acids, including released amounts undetectable by GC–MS, while still allowing intracrine FFA4 activation. The presence of 10 μM BSA in the culture medium completely abolished mGα_i/o_ recruitment to FFA4 in response to stimulation with α-linolenic acid concentrations as high as 10 μM while only marginally influencing FFA4 activation in response to isoproterenol or forskolin, consistent with our hypothesis (Fig. 3f).

Altogether, these results indicate that activation of cell surface receptors by FFAs released into the culture medium cannot explain the rapid FFA4 activation observed in response to pharmacological lipolysis induction, strongly supporting an intracrine mode of action.

### Intracrine FFA4 signaling modulates lipolysis in adipocytes

**Fig. 4:**
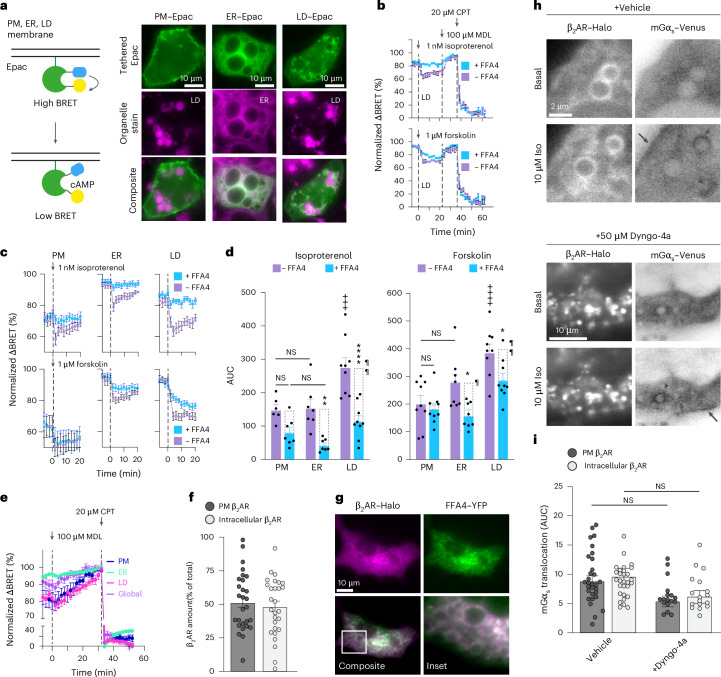
FFA4 exerts a local control over cAMP levels near LDs. **a**, BRET approach to monitor local cAMP changes. Left, schematic of the Epac cAMP sensor (Nluc-Epac-VV). Right, representative HILO images of PM-tethered, ER-tethered or LD-tethered Epac sensor transfected in differentiated immortalized brown adipocytes. LDs and the ER were stained with LipidSpot and ER-Tracker, respectively. **b**, BRET cAMP traces in cells expressing LD-Epac sensor with (+FFA4) or without (−FFA4) FFA4 cotransfection, stimulated with isoproterenol (top) or forskolin (bottom). At the end of each experiment, the cells were treated with an adenylyl cyclase inhibitor (MDL-12330A, MDL) followed by a cell-permeable cAMP analog (8-pCPT-2-*O*-Me-cAMP-AM, CPT) for normalization. **c**, Normalized BRET traces obtained with PM-tethered, ER-tethered and LD-tethered Epac sensors. **d**, Corresponding AUC values. **e**, BRET measurements of basal adenylyl cyclase activity. Cells were transfected with either nontethered (global) or tethered Epac sensor and treated with MDL. **f**, Percentage of β_2_-adrenergic receptor (β_2_AR–Halo) localized at the PM versus intracellular membranes in differentiated immortalized brown adipocytes. **g**, Colocalization between FFA4–YFP and β_2_AR–Halo in differentiated immortalized brown adipocytes. White indicates colocalization. **h**, Venus–mGα_s_ recruitment to β_2_AR–Halo following isoproterenol stimulation in the absence (top) or presence (bottom) of Dyngo-4a to block receptor internalization. The lookup table (LUT) is inverted to facilitate visualization of Venus–mGα_s_ translocation. Arrows, PM; arrowheads, LDs. **i**, Corresponding quantification of mGα_s_ translocation to β_2_AR at the PM or intracellular compartments. Data are the mean ± s.e.m. of *n* = 7 (isoproterenol, PM and ER; forskolin, ER), 9 (isoproterenol, LD; forskolin, PM and LD) (**b**–**d**), 4 (ER) and 6 (PM, LD and global) (**e**) independent biological replicates, *n* = 28 cells (**f**) and *n* = 30 and 17 cells (**i**). Images are representative of *n* = 2 (**a**,**g**) and 12 (**h**) independent experiments. Results in **d** were determined to be statistically significant according to a two-way ANOVA followed by Šidák’s multiple-comparison test. ****P < 0.0001, **P < 0.01 and *P < 0.05 versus corresponding −FFA4 condition; ++++*P* < 0.0001 and +++*P* < 0.001 versus PM −FFA4 condition. ¶¶*P* < 0.01 and ¶*P* < 0.05 versus FFA4-dependent reduction at PM (dashed gray boxes). Source data

We next sought to compare the inhibitory effects of FFA4 on cAMP signaling at the PM versus intracellular sites in adipocytes. For this purpose, we transfected differentiated immortalized brown adipocytes, which express endogenous β-adrenergic receptors and adenylyl cyclases, with a BRET sensor for cAMP, Nluc-Epac-VV^35^, which we tethered to the PM, ER membrane or LD membrane through fusion to targeting domains derived from PDE2A3 (ref. ^36^), Sec61β (ref. ^37^) or PLIN1 (ref. ^38^), respectively (Fig. 4a). Tethered cAMP sensors have been extensively used to measure local cAMP concentrations^39^. While such approaches likely underestimate local cAMP differences as they cannot measure the true cAMP nanodomains that exist very close to cAMP effectors and do not directly determine the source of cAMP production, they can reveal relative, average differences between subcellular compartments^39^. Cells were stimulated with submaximal concentrations of either isoproterenol (1 nM) or forskolin (1 μM) to favor localized cAMP responses. At the end of each experiment, the cells were treated with an adenylyl cyclase inhibitor (MDL-12330A) followed by a saturating concentration of a cell-permeable cAMP analog (8-pCPT-2-*O*-Me-cAMP-AM), which we used to normalize the BRET values to the sensor’s dynamic range and, thus, precisely compare cAMP levels across compartments^40^. Both isoproterenol and forskolin treatments induced a rapid reduction in BRET at all compartments (Fig. 4b–d), indicative of local cAMP increases, which were unexpectedly more pronounced at LDs than at the PM (Fig. 4c,d). Importantly, cotransfection of FFA4 significantly reduced the cAMP responses at the ER and LDs but not at the PM (Fig. 4d), consistent with G_i/o_-mediated inhibition of an intracellular adenylyl cyclase pool. Intriguingly, FFA4 overexpression had a relatively larger inhibitory effect after isoproterenol compared to forskolin, suggesting a potentially larger overlap of the adenylyl cyclase pools accessible to β-adrenergic receptors and FFA4.

**Extended Data Fig. 7:**
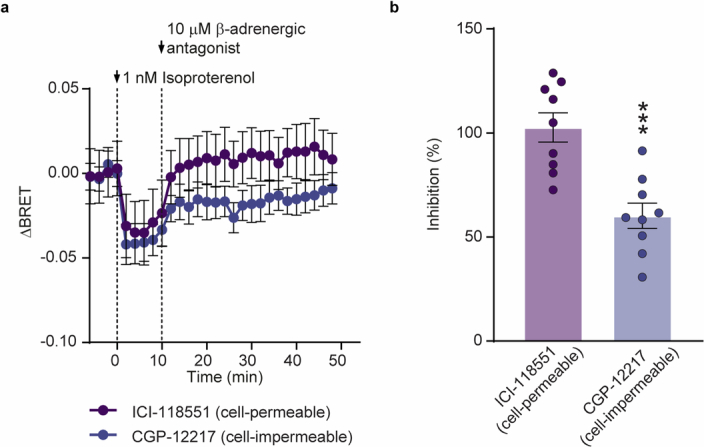
Effect of cell-permeable vs impermeable β-adrenergic antagonists on the isoproterenol-induced cAMP response in differentiated immortalized brown adipocytes. **a**, BRET cAMP traces in cells expressing endogenous β-adrenergic receptors transfected with the non-tethered (global) Epac sensor. Cells were stimulated with isoproterenol, followed by treatment with either a cell-permeable (ICI-118551) or impermeable (CGP-12217) β-adrenergic antagonist. **b**, Corresponding quantification of inhibition. Data are mean ± SEM of n = 9 independent biological replicates. Results in **b** are statistically significant by two-tailed Mann-Whitney test. ***p < 0.001. Source data

We then investigated the potential mechanisms allowing FFA4 to inhibit the intracellular adenylyl cyclase pool. Treatment with the adenylyl cyclase inhibitor MDL-12330A reduced cAMP levels both globally, as measured by an untethered cAMP BRET sensor, and locally at both LDs and ER (Fig. 4e), indicative of basal, constitutive adenylyl cyclase activity. Additionally, HILO experiments revealed that, on average, ~50% of transfected Halo-tagged β_2_-adrenergic receptors are located intracellularly (Fig. 4f), where they colocalize with FFA4 (Pearson’s coefficient = 0.80 ± 0.05), showing a similar pattern surrounding LDs (Fig. 4g). Isoproterenol induced mGα_s_ translocation to both β_2_-adrenergic receptors at the PM and intracellular membranes (Fig. 4h,i). The latter could not be prevented by pretreatment with the dynamin inhibitor Dyngo-4a (ref. ^41^) (Fig. 4h,i), ruling out the involvement of receptor internalization. Additionally, the cAMP response evoked by isoproterenol in differentiated immortalized brown adipocytes expressing endogenous β-adrenergic receptors could be virtually completely blocked by a cell-permeable (ICI-118551) but not a cell-impermeable (CGP-12217) β-adrenergic antagonist (Extended Data Fig. 7). Altogether, these findings are consistent with the presence of a preactivated intracellular adenylyl cyclase pool and an intracellular pool of endogenous β-adrenergic receptors that can be activated by isoproterenol.

**Fig. 5:**
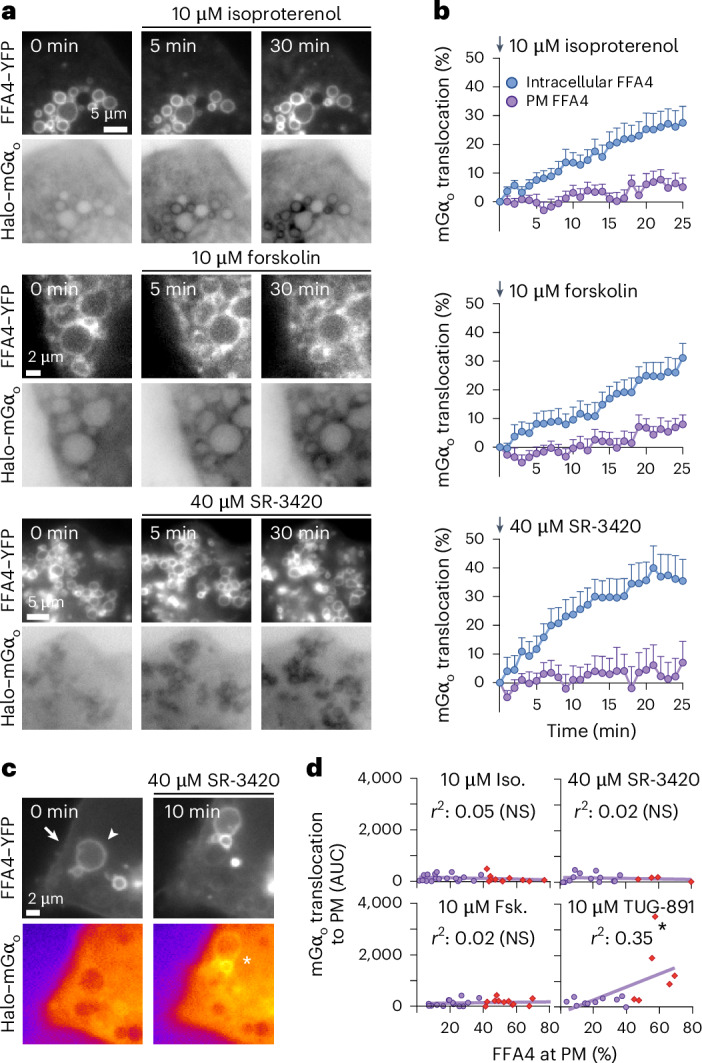
The intracellular pool of FFA4 associated with LDs is rapidly activated upon lipolysis induction. **a**, Representative time courses of Halo–mGα_o_ recruitment to FFA4–YFP in differentiated immortalized brown adipocytes. Cells were stimulated with isoproterenol, forskolin or SR-3420. The Halo–mGα_o_ LUT is inverted to facilitate visualization of Halo–mGα_o_ translocation. **b**, Corresponding quantifications. **c**, Example of a cell with FFA4 at both cell surface and surrounding LDs, showing Halo–mGα_o_ recruitment to the LD-associated compartment but not the PM. Arrow, FFA4 at PM; arrowhead, FFA4 surrounding LDs; star, LD mGα_o_ translocation. **d**, Correlations between mGα_o_ translocation to PM and FFA4 percentage at PM. Red diamonds, additional cells with higher percentage of FFA4 at PM. Data are the mean ± s.e.m. of *n* = 19, 27 and 14 cells (**b**) and *n* = 29, 26, 17 and 16 cells (**d**). Images are representative of *n* = 15 (**a**,**c**) independent experiments. Results in **d** were fitted with a linear regression model. **P < 0.01 versus zero slope according to *F*-test. Source data

**Extended Data Fig. 8:**
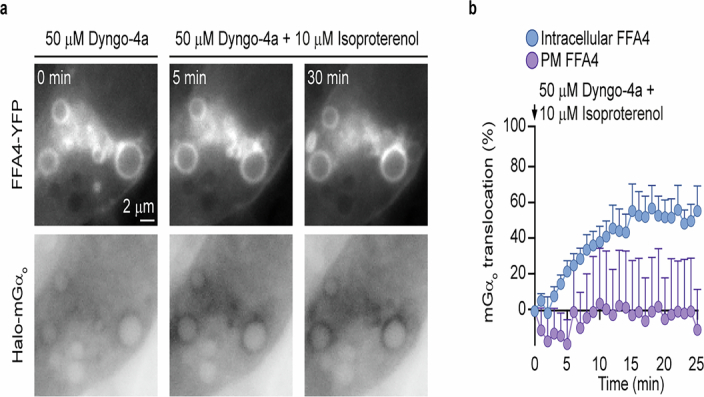
Lipolysis-induced FFA4 activation near LDs does not require receptor internalization. **a**, Representative HILO image sequence of Halo-mGα_o_ recruitment to FFA4-YFP in differentiated immortalized brown adipocytes pre-treated with Dyngo-4a to block receptor internalization, and stimulated with isoproterenol. The Halo-mGα_o_ LUT is inverted to facilitate visualization of Halo-mGα_o_ translocation. **b**, Corresponding quantification. See Fig. 5a,b for comparison. Data are mean ± SEM of n = 4 cells. Images in **a** are representative of 4 independent experiments. Source data

Furthermore, we sought to directly visualize the subcellular location of FFA4 activation following lipolysis induction by performing HILO microscopy experiments in differentiated immortalized brown adipocytes cotransfected with FFA4–YFP and Halo–mGα_o_. Remarkably, lipolysis induction with isoproterenol, forskolin or SR-3420 caused a rapid and robust translocation of Halo–mGα_o_ to the intracellular FFA4 pool located in close proximity to LDs, which was detectable less than 5 min after stimulation; in contrast, virtually no PM translocation was observed (Fig. 5a–c and Supplementary Video 1). The isoproterenol-induced Halo–mGα_o_ translocation to the intracellular FFA4 pool could not be prevented by Dyngo-4a (Extended Data Fig. 8), ruling out that it was because of internalization and trafficking of FFA4 from the PM.

While FFA4 was predominantly located intracellularly in most well-differentiated cells, we extended these experiments to deliberately include an additional group of less differentiated cells with relatively high PM FFA4 levels, which allowed us to correlate the degree of FFA4 PM activation with its PM localization (Fig. 5d, red diamonds). As expected, a significant correlation was observed in cells stimulated with TUG-891, with clearly detectable PM responses in cells with higher PM FFA4 levels (Fig. 5d). By contrast, no correlation was observed in the case of isoproterenol, forskolin or SR-3420, where virtually no PM translocation was observed also in cells with high PM FFA4 levels (Fig. 5d). These results further indicate that the FFAs released following lipolysis preferentially activate FFA4 at intracellular sites, irrespective of the amount of FFA4 at the PM.

**Fig. 6:**
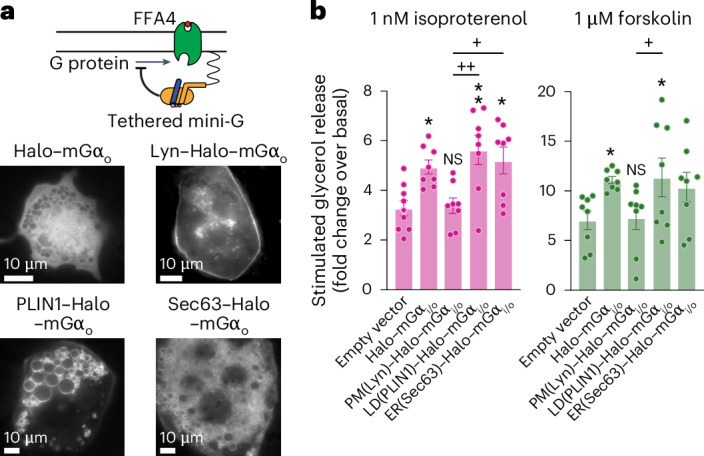
Interfering with G_i/o_ signaling on LD and ER membranes enhances lipolysis. **a**, Local inhibition of G-protein signaling with overexpression of tethered mini-G proteins. Top, schematic depicting local inhibition of G-protein binding to active FFA4 by a tethered mini-G protein. Bottom, representative HILO images of untethered Halo–mGα_o_, or Halo–mGα_o_ tethered to the PM (through Lyn), LDs (through PLIN1) or ER (through Sec63). **b**, Effect of local inhibition of G_i/o_ signaling with tethered Halo–mGα_i/o_ proteins on lipolysis. Differentiated immortalized brown adipocytes were either mock-transfected (empty vector) or cotransfected with untethered Halo–mGα_i/o_ or Halo–mGα_i/o_ tethered to the PM (Lyn), LDs (PLIN1) or ER (Sec63). Data are the mean ± s.e.m. of *n* = 8 independent biological replicates (**b**). Images are representative of *n* = 2 (**a**) independent experiments. Results in **b** were determined to be statistically significant according to a one-way ANOVA followed by Šidák’s multiple-comparison test. **P < 0.01 and *P < 0.05 versus empty vector; ++*P* < 0.01 and +*P* < 0.05. Source data

**Extended Data Fig. 9:**
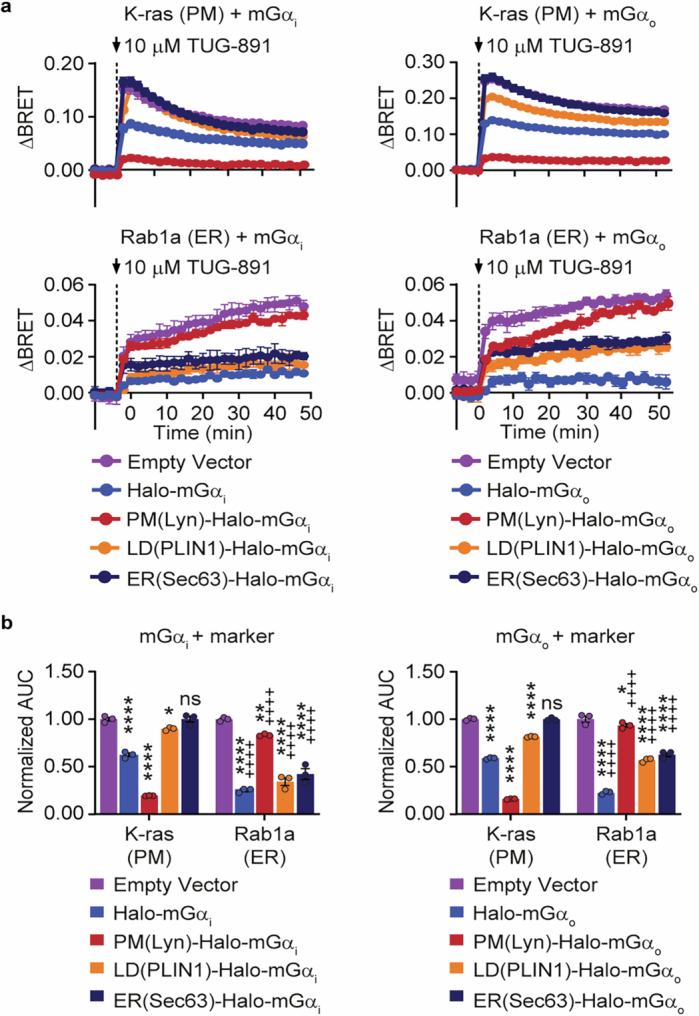
Mini-G proteins tethered to ER and LD membranes specifically inhibit local mini-G protein recruitment. **a**, Effect of PM (Lyn), ER (Sec63) or LD (PLIN1)-tethered Halo-mGα_i/o_ overexpression on agonist-dependent translocation of mGα_i_ (left) or mGα_o_ (right) to Venus-tagged K-ras (PM) (top) or Rab1a (ER) (bottom), monitored by real-time bystander BRET. Untethered Halo-mGα_i/o_ is included for comparison. **b**, Corresponding normalized AUC values. Data are mean ± SEM of n = 3 independent biological replicates. Results are statistically significant by two-way ANOVA followed by Dunnett’s post-hoc test. ****p < 0.0001, **p < 0.01, *p < 0.05 vs corresponding empty vector. ^++++^p < 0.0001 vs corresponding K-ras (PM). ns, statistically not significant. Source data

Lastly, we attempted to evaluate the consequences of FFA4 signaling at intracellular sites on lipolysis. For this purpose, we overexpressed mini-G proteins tethered to the PM, LDs or ER through fusion to specific targeting domains (PM, Lyn; LDs, PLIN1; ER, Sec63) as local inhibitors of G_i/o_ coupling (Fig. 6a). Experiments in HEK293T cells (Extended Data Fig. 9) confirmed that Sec63-tethered and PLIN1-tethered Halo–mGα_i/o_ inhibited G_i/o_ signaling at the ER (58%/38% and 66%/43% reductions, respectively), while having negligible (Sec63) or only modest (PLIN1, 10%/18%) effects at the PM. By contrast, Lyn-tethered Halo–mGα_i/o_ strongly inhibited G_i/o_ signaling at the PM (81%/84% reduction), while only modestly affecting signaling at the ER (16%/6% reduction). As a control, untethered Halo–mGα_i/o_ inhibited both ER and PM signaling as expected (Extended Data Fig. 9). Once transfected in immortalized brown adipocytes, all constructs showed the desired localization (Fig. 6a). Overexpression of LD (PLIN1)–Halo–mGα_i/o_ in immortalized brown adipocytes significantly enhanced lipolysis in response to both isoproterenol (71% ± 10%) and forskolin (61% ± 17%) (Fig. 6b). A similar enhancement was observed with ER (Sec63)–Halo–mGα_i/o_ (58% ± 10% and 47% ± 14% for isoproterenol and forskolin, respectively), albeit statistically significant only following isoproterenol stimulation. By contrast, PM (Lyn)–Halo–mGα_i/o_ had no statistically significant effects on lipolysis induced by either isoproterenol or forskolin. These results, obtained in cells expressing endogenous adrenergic receptors, adenylyl cyclases and FFA4, suggest that FFA4 signaling from the LD-associated compartment is required for efficient lipolysis inhibition. Moreover, the somewhat stronger effect observed with PLIN1-tethered compared to Sec63-tethered Halo–mGα_i/o_ is consistent with FFA4 signaling from ER subdomains associated with LDs as opposed to the entire ER.

Altogether, these findings support a model whereby, upon lipolysis induction, the endogenously released FFAs rapidly bind to and activate an intracellular FFA4 pool that resides in close proximity to LDs, where they locally couple to G_i/o_ to inhibit cAMP signaling and modulate lipolysis in an intracrine fashion.

## Discussion

**Extended Data Fig. 10:**
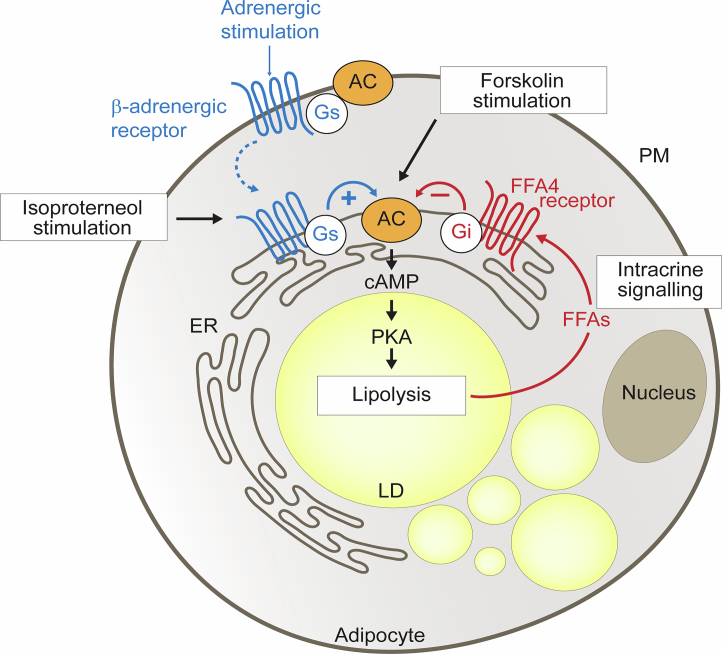
Cartoon summarizing the new paradigm of ‘intracrine’ FFA4 signaling at LDs as proposed by the present study. Adrenergic stimulation activates the lipolytic machinery located at the surface of a LD via stimulation of the Gs/cAMP/PKA signaling pathway, with consequent local release of FFAs. The released FFAs rapidly bind to an intracellular FFA4 pool that resides on membranes of the ER intimately associated with the LD. This intracellular FFA4 pool, in turn, inhibits via G_i/o_ signaling local, active adenylyl cyclases, reducing local cAMP production and, ultimately, lipolysis. This provides a previously unknown ‘intracrine’ negative feedback mechanism that allows a LD to rapidly and precisely sense the FFAs released from its own store and adjust its metabolic flux accordingly.

Our study on FFA4 in adipocytes suggests an unanticipated scenario whereby a metabolite-sensing GPCR is located and signals close to the intracellular site where its endogenous ligand is produced, thus modulating a key metabolic pathway in a local fashion (Extended Data Fig. 10). These results not only provide insights into the mechanisms used by FFA4 to rapidly regulate lipolysis but also provide evidence for the occurrence of an intracrine modality of GPCR signaling, which is conceptually distinct from known mechanisms of endocrine, paracrine and autocrine signaling.

For more than 40 years, FFAs released from adipocytes have been known to inhibit lipolysis^11^; however, the underlying mechanisms have been unknown. Recently, our team provided evidence that the antilipolytic effects of FFAs are at least partially mediated by FFA4 through a negative feedback loop^11^. In undifferentiated 3T3-L1 cells, we previously observed FFA4 coupling to G_i/o_, which could explain its antilipolytic effects^11^. However, the mechanisms involved in the FFA4 negative feedback loop in differentiated adipocytes and, importantly, its subcellular location were not investigated. This study provides insights into these fundamental mechanisms that have a key role in physiology and disease and could extend to other metabolite-sensing GPCRs.

First, we show that both endogenous FFA4 in native mouse tissue and fluorescently tagged FFA4 expressed in differentiated immortalized adipocytes are predominantly localized intracellularly. In differentiated immortalized adipocytes, FFA4, but not HCA2 used as a control, is unexpectedly present on membranes that are intimately associated with LDs, most likely ER subdomains.

Second, we provide evidence that, despite being generally classified as a G_q_-coupled receptor^12,13^, FFA4 is mainly coupled to G_i/o_ in differentiated adipocytes, which explains its rapid negative effects on lipolysis through inhibition of intracellular cAMP signaling. While our results highlight the importance of G_i/o_ signaling in FFA4-dependent lipolysis regulation, they do not exclude a contribution of G_q_ (ref. ^12^) or arrestin^13^ signaling to other biological effects of FFA4. For instance, in addition to being involved in the FFA4-dependent stimulation of glucose uptake in adipocytes, G_q_ coupling is known to modulate incretin release in enteroendocrine cells^12^.

Third, our results suggest that endogenous FFAs released during lipolysis can rapidly activate the LD-associated FFA4 pool, allowing it to locally inhibit cAMP production through G_i/o_ signaling, which we show to be required for FFA4 to efficiently modulate lipolysis.

The proposed occurrence of G-protein signaling at the ER is in keeping with the well-known assembly of G proteins on ER membranes, from where they reach the PM through anterograde trafficking^42,43^. Similarly, adenylyl cyclases are synthesized in the ER and have been shown to be abundant on its membranes^44^. Intriguingly, a recent proteomic study also detected the presence of G proteins, including Gα_s_, Gα_i2_, Gα_q_ and Gβ_3_ subunits in LD fractions^45^. Our results substantially expand these previous observations by indicating that, in addition to the whole machinery required for G-protein signaling and cAMP production, FFA4 is present and active in the vicinity of LDs, providing a canonical, receptor-dependent mechanism to locally modulate cAMP signaling at this key intracellular compartment.

In accordance with FFA4-mediated inhibition of cAMP signaling from LD-associated membranes, our study suggests the presence of an intracellular pool of active adenylyl cyclases. Several mechanisms are likely to contribute to its activation. First, our data are consistent with the presence of basal constitutive adenylyl cyclase activity, which can be further increased by cell-permeable forskolin. Moreover, they suggest that isoproterenol, although generally considered to be membrane impermeable, can induce internalization-independent activation of intracellular β-adrenergic receptors and G_s_ coupling near LDs in adipocytes. This is consistent with a previous report that isoproterenol can activate intracellular β_1_-adrenergic receptors in cardiomyocytes^46^ but not with another study indicating that it can only activate cell surface receptors^47^. While further studies will be required to reconcile such apparent discrepancies between previous studies and potential differences among cell types, one possible explanation might lie in the differential expression of transporters capable of facilitating isoproterenol diffusion across the PM.

The apparent localization of FFA4 on ER membranes surrounding LDs and the fact that PLIN1-tethered mini-G_i/o_ proteins are particularly effective at inhibiting agonist-induced lipolysis are consistent with the well-known notion that LDs emanate directly from ER membranes and maintain close contact with them^25^. Related to this, there are two mechanisms responsible for protein targeting to LDs, that is, lateral diffusion from ER to LD membranes (class I) and binding from the cytoplasm (class II), which could both explain the observed inhibition of ER-localized FFA4 by PLIN1-tethered mini-G_i/o_. In fact, while PLINs are often considered class II proteins, recent data suggest that PLIN1 might also associate with ER membranes and reach LDs through lateral diffusion from the ER, behaving essentially as a class I protein^25^.

Of note, our finding that intracellular FFA4 modulates lipolysis does not exclude that cell surface FFA4 can also contribute to the regulation of adipocyte functions. On the contrary, these two FFA4 pools are likely to serve different and potentially synergistic functions, such as the sensing of extracellular and intracellular FFAs. This view is consistent with the fact that fatty acids bind to specific carrier proteins both extracellularly, above all albumin^48^, and intracellularly (that is, FABPs^49^), which limit their diffusion and regulate their local ‘free’ concentrations. In this context, the apparent shift of FFA4 subcellular localization from the PM in undifferentiated cells to intracellular sites in differentiated adipocytes may be part of the highly regulated adipocyte maturation program associated with LD biogenesis. In agreement with this hypothesis, FFA4 expression is strongly induced during adipocyte maturation^50,51^ and has been shown to promote LD biogenesis^51,52^. Moreover, *FFAR4* is one of the genes most strongly upregulated by exposure to cold in BAT^53^. Future studies will be required to investigate the complex interplay between the mechanisms that control FFA4 expression, its subcellular localization and the formation of LDs during adipocyte differentiation.

Importantly, previously described GPCR signaling mechanisms, including ‘endosomal’ GPCR signaling, involve the secretion of a mediator into the extracellular space and its action at a distance. In contrast, our study suggests a new mechanism whereby a GPCR appears to function as an intracellular sensor for an intracellular metabolite allowing a cell or even a single subcellular organelle such as a LD to rapidly modify its metabolism without the need for the metabolite to be released and accumulate in the extracellular space.

Given the potential of FFA4 as a drug target for the treatment of metabolic and inflammatory disease^17,26^, our findings could also have important therapeutic implications. Above all, they indicate that drugs exploiting antilipolytic effects of FFA4 should cross the PM and access the intracellular FFA4 pool associated with LDs to efficiently modulate lipolysis. Moreover, the fact that FFA4 appears to signal from multiple subcellular compartments that differ among cell types opens up the possibility of selectively activating or inhibiting FFA4 signaling at specific subcellular locations to induce more specific pharmacological responses while minimizing unwanted effects.

Whereas our study supports such a model of intracrine FFA4 signaling, it has some limitations. First, while we demonstrate that lipolysis-induced fatty acid release can activate intracellular FFA4, as evidenced by mini-G translocation, we cannot exclude the possibility of an alternative intracrine mechanism whereby intracellularly released fatty acids might activate cell surface FFA4 through the cytosol. Second, despite advances in RET techniques and cAMP biosensors, precisely localizing the source of cAMP production within living cells remains challenging. While our cAMP BRET results are compatible with the proposed model whereby LD/ER-associated FFA4 would inhibit a local adenylyl cyclase pool, alternative explanations, including potential mechanisms involving the participation of receptors and/or adenylyl cyclases at the PM, cannot be completely ruled out. In this respect, although endogenous β_2_-adrenergic activation appears to cause larger cAMP increases at LD/ER than at the PM and overexpressed β_2_-adrenergic receptors localize to and can be activated by isoproterenol on intracellular membranes in adipocyte cell lines, further studies are needed to determine the exact localization of endogenous adrenergic receptors and their mechanism of activation under physiological conditions. These experiments will hopefully also resolve the still open question about the possible transport of endogenous adrenergic agonists across biological membranes. Similarly, additional studies appear required to directly localize the adenylyl cyclase pools present in adipocytes and further clarify the mechanisms involved in their regulation by both adrenergic receptors and FFA4, which was only partially addressed in our study. Lastly, additional studies will be needed to better clarify the relevant contribution of the PM versus intracellular FFA4 pool to the regulation of lipolysis. These experiments, which will likely require the development of novel and more precise sensors and approaches, will hopefully provide a more comprehensive picture about the mechanisms involved in the physiological regulation of lipolysis in adipocytes and the role of the proposed model of intracrine FFA4 signaling.

Altogether, our study points to the occurrence of a previously unrecognized intracrine signaling modality by a prototypical metabolite-sensing GPCR. Moreover, it uncovers the existence of a specialized intracellular signaling hub intimately associated with LDs, which provides a mechanism to rapidly and efficiently regulate lipolysis in adipocytes.

## Methods

### Chemicals and reagents

TUG-891 (SML1914), MDL-12,330A hydrochloride (M182), α-linolenic acid (L2376), atglistatin (SML1075), poly(d-lysine) hydrobromide (P7886), human insulin (I9278), 3,3′,5-triiodo-l-thyronine (T3) sodium salt (T6397), dexamethasone (D4902), 3-isobutyl-1-methylxanthine (IBMX; I5879), bovine insulin (I0516), palmitoleic acid (P9417), oleic acid (O1008), palmitic acid (P0500), HEPES (H0887), sodium deoxycholate (D6750), igepal (I3021), *N*,*N*-diisopropylethylamine (D125806), pentaflurobenzylbromine (101052), 2× Laemmli sample buffer (53401), goat serum (G9023), Krebs ringer buffer (K4002), dichloromethane (650463), butylated hydroxytoluene (W218405) and heptadecanoate acid (H3500) were obtained from Merck Sigma-Aldrich. ER-Tracker (E34250), uncoupling protein 1 (UCP1) polyclonal antibody (PA1-24894), PLIN1 monoclonal antibody (MA5-27861), goat anti-rabbit secondary antibody conjugated with Alexa Fluor 488 (A-11008), goat anti-rat secondary antibody conjugated with biotin (31830), Pierce high-sensitivity streptavidin–HRP (horseradish peroxidase; 21130), DMEM (11584486), high-glucose GlutaMAX DMEM (12077549), FluoroBrite phenol red-free DMEM (15266695), FBS (10500064), bovine calf serum (11551831), penicillin and streptomycin (15140122), l-glutamine (25030), 0.05% trypsin–EDTA (25300054), Dulbecco’s PBS (DPBS; 1419094), Hanks’ balanced salt solution (HBSS; 14025092), paraformaldehyde (11586711), BCA assay (23229), 4–12% Bis–Tris SDS–PAGE gradient gels (NP0321BOX), nitrocellulose membrane (88018), polyethyleneimine (040527) and Lipofectamine 2000 (11668500) were purchased from Thermo Fisher Scientific. IRDye donkey anti-rabbit secondary antibody (926-32213) and donkey anti-rat secondary antibody (926-32219) were from LI-COR Biotechnology. Rabbit anti-Na^+^,K^+^-adenosine triphosphatase (ATPase) antibody (ab76020) was from Abcam. HA monoclonal antibody (11867423001), protease inhibitor cocktail (5892970001) and anti-HA affinity matrix beads (11815016001) were from Roche. AH7614 (5256) and YM-254890 (7352) were from Bio-Techne. Isoproterenol hydrocholoride (1747), 8-pCPT-2-*O*-Me-cAMP-AM (4853), ICI-118551 (0821) and CGP-12217 (1134) were from Tocris. Forskolin (11018), rosiglitazone (71740) and GC–MS fatty acid standards (17942) were from Cayman Chemical. Water (232178) and methanol (136841) for GC–MS were from BioSolve. Nano-Glo luciferase assay substrate (N1120), HaloTag Janelia Fluor 646 (GA1120) and GloSensor cAMP assay (E1290) were from Promega. SNAP-Cell 647 (S9102S), BamHI (R0136), EcoRI (R3101), AsiSI (R0630) and XhoI (R0146) were from New England Biolabs. Hypergrade acetonitrile (1.00029) and GC–MS-grade SupraSolv isooctane (1.15440) were from Supelco. Prolume purple II coelenterazine (367) was from NanoLight Technology. LipidSpot (70069) was purchased from Biotum. Succinylated WGA agarose bound beads (AL-1023S) and ProLong Diamond antifade mountant (H-1000) were from Vector Laboratories. BAY 59-9435 (HY-102056) was from MedChem Express. The TransIT-X2 dynamic delivery system (MIR 6000) was from Mirus. Pertussis toxin (PHZ1174) was from Invitrogen. Dyngo-4a (B5997) was from Stratech Scientific. Fatty-acid-free BSA (11945.03) was from SERVA. SR-3420 was provided by J. G. Granneman.

### Animals

mFFA4–HA^26^, hFFA2–HA^27^ and CRE-MINUS^27^ (harboring hFFA2–HA at the mouse *Ffar2* locus but without induced expression) mice were generated by genOway. All animals were bred as homozygous onto a C57BL/6 background. FFA4-knockout βGAL mice were provided by AstraZeneca^54^. Breeding, maintenance and killing of mice conformed to Home Office regulations (project license number: PP0894775).

### Plasmids

A plasmid encoding FFA4–eYFP was previously reported^13^. The FFA4 sequence was subcloned into pcDNA3 following BamHI and EcoRI digestion. Nluc was subcloned from mG-Nluc using EcoRI and XhoI restriction sites and ligated into the pcDNA3 FFA4 construct to generate FFA4 containing Nluc fused to its C terminus. To generate a plasmid encoding SNAP-tagged FFA4, the FFA4 sequence from FFA4–eYFP was subcloned into a previously validated N-terminal SNAP-tagged human β_2_-adrenergic receptor construct^55^ using BamHI and XhoI digestion. All tagged FFA4 constructs were functional and induced signaling profiles comparable to untagged FFA4 (Extended Data Fig. 2). Halo was subcloned from ScFv30–Halo^55^ using BamHI and XhoI restriction sites and ligated into the SNAP–β_2_-adrenergic construct^55^ to generate a SNAP–β_2_-adrenergic–Halo construct. A plasmid containing HCA2 was purchased from Origene. The eYFP sequence was obtained from an eYFP construct using XhoI and AsiSI restriction sites and ligated into the HCA2 construct to generate HCA2 containing eYFP fused to its C terminus (HCA2–eYFP). Plasmids encoding mini-G probes^16^ were provided by N. Lambert. Plasmids encoding Sec63-tagged and PLIN1-tagged Halo–mGα_i_ and Halo–mGα_o_ probes were generated by gene synthesis (Twist Bioscience). A PLIN1 construct^56^ was provided by D. Savage. A plasmid encoding Halo–Sec61β (ref. ^37^) was a gift from C. Obara and Rab5–mCherry were provided by T. Kirchhausen. Plasmids expressing Venus-tagged subcellular markers^18^ were provided by K. Pfleger and a plasmid encoding the Nluc-Epac-VV BRET sensor^35^ was provided by K. Martemyanov. A plasmid encoding Nluc-Epac-VV with Sec61β (ref. ^37^) fused to its C terminus was cloned by PCR and Gibson assembly using Nluc-Epac-VV as a template. Likewise, plasmids encoding Nluc-Epac-VV carrying a PDE2A3 PM-targeting domain^41^ or full-length PLIN1 (ref. ^43^) at the N terminus were cloned by PCR and Gibson assembly using Nluc-Epac-VV as a template. Plasmids encoding Gα_i1_ (ref. ^15^), Rap1GAP–RlucII (ref. ^15^), rGFP–CAAX^19^, rGFP–FYVE^19^, tdrGFP–Giantin^20^ and tdrGFP–PTP1B (ref. ^20^) were previously reported.

### Cell culture and transfection

HEK293T cells (American Type Culture Collection (ATCC), CRL3216) were cultured in DMEM supplemented with 10% FBS, 100 U ml^−1^ penicillin and 0.1 mg ml^−1^ streptomycin at 37 °C, 5% CO_2_. HEK293SL (ref. ^19^) cells were cultured in DMEM supplemented with 10% FBS and 1% penicillin and streptomycin at 37 °C, 5% CO_2_. For BRET and GloSensor cAMP assay experiments in HEK293T cells, cells were plated onto six-well plates at a density of 7 × 10^5^ cells per well. The next day, they were transfected with Lipofectamine 2000 following the manufacturer’s protocol. After 24 h, cells were resuspended in FluoroBrite phenol red-free DMEM medium supplemented with 4 mM l-glutamine and 5% FBS and plated onto poly(d-lysine)-coated 96-well white polystyrene Nunc microplates (165306) at a density of 1 × 10^5^ cells per well and allowed to adhere for 24 h. For live-cell imaging, HEK293T cells were seeded onto 25-mm round glass coverslips (VWR, 631-1584) at a density of 5 × 10^5^ cells per well. The next day, they were transfected with Lipofectamine 2000, following the manufacturer’s protocol. Cells were imaged 24 h after transfection. For ebBRET experiments in HEK293SL cells, cells were seeded into 96-well plates at a density of 3.5 × 10^4^ cells per well and transfected simultaneously with polyethyleneimine. Measurements were performed 48 h after transfection.

Immortalized mouse brown preadipocytes^22,57^ were cultured in high-glucose GlutaMAX DMEM supplemented with 10% FBS, 100 U ml^−1^ penicillin and 0.1 mg ml^−1^ streptomycin at 37 °C, 5% CO_2_. Preadipocytes were seeded onto 10-cm dishes at a density of 1.3 × 10^6^ cells per dish, 96-well plates at a density of 6 × 10^4^ cells per well or 25-mm round glass coverslips at a density of 5 × 10^5^ cells per well for BRET measurements, glycerol quantification assays and imaging experiments, respectively. After 48–72 h, the medium was replaced with complete high-glucose GlutaMAX DMEM containing 500 μM IBMX, 1 μM dexamethasone, 1 nM T3, 0.5 μM rosiglitazone and 20 nM human insulin for 48 h, followed by complete high-glucose GlutaMAX DMEM containing 1 nM T3 and 20 nM human insulin for 24 h to induce differentiation. The following day, differentiated adipocytes were transfected using the TransIT-X2 transfection reagent as per the manufacturer’s protocol. For BRET assays, differentiated immortalized brown adipocytes grown on 10-cm dishes were detached with 0.05% trypsin–EDTA, reverse-transfected and replated onto Nunc microplates at a density of 7.5 × 10^4^ cells per well in high-glucose GlutaMAX DMEM supplemented with 10% FBS; for imaging experiments, differentiated immortalized brown adipocytes grown on 25-mm round glass coverslips were forward-transfected. All transfected adipocytes were incubated for 24 h before being used for BRET assays or imaging.

The 3T3-L1 cells (ATCC, CL-173) were cultured in DMEM supplemented with 10% bovine calf serum, 100 U ml^−1^ penicillin and 0.1 mg ml^−1^ streptomycin at 37 °C, 5% CO_2_. Undifferentiated 3T3-L1 cells were seeded onto 10-cm dishes and, once confluent, transfected by electroporation. Confluent cells were detached with 0.05% trypsin–EDTA, pelleted and resuspended in 240 μl of DPBS, followed by the addition of 40 μg of DNA into 4-mm cuvettes. Cells were electroporated at 320 V and 125 μF using a Gene Pulser XCell eukaryotic system (Bio-Rad) and resuspended in 1 ml of DMEM supplemented with 10% bovine calf serum, 100 U ml^−1^ penicillin and 0.1 mg ml^−1^ streptomycin. Subsequently, 100 μl of the suspension containing transfected cells was plated onto 25-mm round glass coverslips and allowed to adhere. To induce differentiation, the medium was replaced 48 h later with DMEM supplemented with 10% FBS, 1 μM dexamethasone, 0.5 mM IBMX, 1.0 μg ml^−1^ bovine insulin, 100 U ml^−1^ penicillin and 0.1 mg ml^−1^ streptomycin and left for 48 h. The medium was replaced every 48 h until cells reached the desired differentiation (7–15 days).

### BRET assays

On the day of the experiment, the medium was replaced with HBSS containing 10 mM HEPES pH 7.5 and 10 μM furimazine with Nano-Glo Luciferase assay substrate. BRET measurements were performed at 37 °C using a PHERAstar FSX Microplate Reader (BMG Labtech) with Mars data analysis software version 3.32 and a dual-luminescence BRET1 plus readout filter set (460–490-nm and 520–550-nm emission filters). Following four baseline measurements, the cells were treated with vehicle or the indicated agonist concentration and measured for an additional hour. BRET acceptor–donor ratios were calculated separately for each well. Shown are the changes in BRET ratio over basal after vehicle subtraction (ΔBRET). Measurements were performed using at least two technical replicates.

### ebBRET assay

On the day of the experiment, HEK293SL cells were washed with PBS followed by Tyrode’s buffer (140 mM NaCl, 2.7 mM KCl, 1 mM CaCl_2_, 12 mM NaHCO_3_, 5.6 mM d-glucose, 0.5 mM MgCl_2_, 0.37 mM NaH_2_PO_4_ and 25 mM HEPES, pH 7.4). The cells were then incubated with Tyrode’s buffer containing 1.3 μM Prolume purple II coelenterazine and the indicated agonist for 10 min. BRET measurements were performed at 37 °C using a Spark multimode microplate reader (Tecan) with a BRET^2^ filter set (365–435-nm and 505–525-nm emission filters). The BRET signal was determined by calculating the ratio of the light intensity emitted by the acceptor over the light intensity emitted by the donor. Data are presented as ligand-promoted BRET (ΔBRET).

### GloSensor cAMP assay

On the day of experiment, the cell medium was replaced with equilibration buffer, consisting of FluoroBrite phenol red-free DMEM medium supplemented with 4 mM l-glutamine, 5% FBS and 2% reconstituted GloSensor cAMP reagent (Promega). Cells were incubated for 2 h at 37 °C, 5% CO_2_. Luciferase measurements were performed at 37 °C using a PHERAstar microplate reader (BMG Labtech). Following four baseline measurements, the cells were treated with vehicle or the indicated agonist concentrations alongside 10 μM forskolin and measured for an additional hour.

### Live-cell protein labeling

Cells were labeled with 1 μM HaloTag Janelia Fluor 646 (JF646) or 1 μM SNAP-Cell 647 (SiR 647) in complete culture medium (without antibiotics) for 20 min at 37 °C. Cells were then washed three times with complete culture medium, allowing a 5-min incubation between washes.

### HILO live-cell imaging

First, 25-mm round glass coverslips were mounted in a microscopy chamber filled with HBSS supplemented with 10 mM HEPES pH 7.5. The sample and objective were maintained at 37 °C using a temperature-controlled enclosure throughout the experiments. Live-cell HILO imaging was performed on a custom total internal reflection fluorescence (TIRF) microscope (assembled by CAIRN Research). The system was based on an Eclipse Ti2 microscope (Nikon) equipped with four electron-multiplying charge-coupled device (EMCCD) cameras (iXon Ultra 897, Andor), ×100 oil-immersion objective (SR HP APO TIRF, numerical aperture: 1.49; Nikon), an iLas2 TIRF illuminator (Gataca Systems), 405-nm, 488-nm, 561-nm and 637-nm diode lasers (Coherent, Obis), a quadruple beam splitter, quadruple band excitation and dichroic filters, a ×1.5 tube lens and hardware focus stabilization. Two of the four synchronized EMCCD cameras were used to acquire simultaneous image sequences at a rate of one image every 30 s. HILO images were acquired with MetaMorph 7.10.2.240.

### SIM

Differentiated immortalized brown adipocyte cells were seeded and transfected on precision cover glasses, thickness no. 1.5H (Marienfeld, CG15NH1). Cells were washed twice with DPBS for 5 min each and fixed with 4% paraformaldehyde in 0.1 M PIPES, 2 mM EGTA and 1 mM MgSO_4_ (pH 6.95) for 15 min at room temperature and quenched with 50 mM ammonium chloride. After fixation, coverslips were air-dried before mounting with ProLong Diamond antifade mountant. Slides were cured at room temperature for 48 h before imaging. Images were acquired using two- and three-dimensional SIM and reconstructed in NIS-Elements (Nikon) or ARIVIS (Zeiss), respectively.

### Image-based quantification of mini-G probe translocation

Ilastik (version 1.4.0)^23^ was used to train a random forest to classify pixels from the receptor channel into three classes: receptor, intracellular background and extracellular background. One classifier was trained for each cell line (that is, 3T3-L1 and immortalized brown adipocytes) and all default image features were used. Training images were selected across conditions and replicates. The remaining analysis was performed in MATLAB. First, the probabilities from the Ilastik classifier were thresholded at 0.5 for the receptor and intracellular background classes to give binary segmentations. Receptor and intracellular background segmentations were then combined (logical OR) to get a binary cell mask. This cell mask was postprocessed by hole filling, erosion and dilation. A ‘PM’ mask was defined as a 7-pixel-deep band at the edge of the cell mask. A ‘cell interior’ mask was defined by excluding the PM mask from the cell mask. The percentages of receptor signal at the PM (overlap with PM mask) and intracellular membranes (overlap with cell interior mask) were then quantified for each time point. To quantify mini-G translocation to receptors at the PM and intracellular membranes, we calculated the densities of mini-G colocalizing with receptors at the PM (overlap with both the binary receptor segmentation and PM mask) and intracellular membranes (overlap with both the binary receptor segmentation and cell interior mask), which were normalized dividing by the cytoplasmic mini-G density. The percentage change (that is, translocation) over time is shown.

### Tissue dissection

Following cervical dislocation, epididymal WAT and intrascapular BAT were removed and immediately placed in cold Krebs–bicarbonate solution containing 118.4 mM NaCl, 24.9 mM NaHCO_3_, 1.9 mM CaCl_2_, 1.2 mM MgSO_4_, 4.7 mM KCl, 1.2 mM KH_2_PO_4_ and 11.7 mM glucose (pH 7.0), supplemented with protease inhibitors.

### Tissue lysate preparation

Tissue lysates were prepared following previous methods^27^. In brief, tissue samples were lysed in radioimmunoprecipitation assay (RIPA) buffer containing 50 mM Tris, 150 mM NaCl, 0.5% sodium deoxycholate, 1% igepal, 0.1% SDS and protease inhibitors. The resulting lysates were passed through a fine needle and centrifuged at 20,000*g* for 15 min at 4 °C. Protein quantification and normalization were performed using a BCA assay.

### WGA and HA-tagged receptor precipitation

Tissue lysates were added to succinylated WGA agarose beads and incubated overnight at 4 °C. WGA agarose beads were separated from the lysate by centrifugation at 2,000*g* for 1 min. WGA complexes were washed in RIPA buffer thrice and incubated in 2× Laemmli sample buffer at 60 °C for 5 min before centrifugation at 20,000*g* for 2 min. The remaining unbound lysate was further immunoprecipitated overnight at 4 °C using anti-HA affinity matrix beads. HA immunocomplexes were centrifuged at 2,000*g* for 1 min and washed thrice with RIPA buffer. HA-tagged proteins were eluted by resuspension in 2× Laemmli sample buffer, followed by incubation at 60 °C for 5 min, and centrifugation at 20,000*g* for 2 min.

### Western blot analysis

Western blot analysis was performed following previous methods^27^. Briefly, 25 μl of sample was loaded onto 4–12% Bis–Tris SDS–PAGE gradient gels. Proteins were separated and transferred onto a nitrocellulose membrane. Nonspecific binding was blocked using 5% BSA in Tris-buffered saline (TBS) supplemented with 0.1% Tween-20 for 1 h at room temperature. Membranes were incubated with primary anti-HA high-affinity antibody from rat IgG1 or primary rabbit anti-Na^+^,K^+^-ATPase antibody (1:1,000 dilution) in TBS containing 0.1% Tween-20 and 5% BSA overnight at 4 °C. The next day, the membranes were washed with TBS supplemented with 0.1% Tween-20 and incubated with secondary donkey anti-rabbit conjugated to IRDye 800CW antibody or secondary donkey anti-rat conjugated to IRDye 800CW antibody (1:10,000 dilution) for 1 h at room temperature. Proteins were visualized using an Odyssey imaging system.

### Immunofluorescence

Immunofluorescence was performed following previous methods^27^. In brief, tissues were fixed in 4% paraformaldehyde and embedded in paraffin wax. Tissues were sectioned (5 µm), deparaffinized and antigen-retrieved. Resulting sections were washed with TBS containing 0.1% Triton X-100 and blocked for 2 h in TBS containing 0.1% Triton X-100, 1% BSA and 3% goat serum at room temperature. Sections were then incubated with primary anti-HA high-affinity antibody from rat IgG1 (1:250 dilution) in combination with either primary rabbit anti-UCP1 (1:500 dilution) or primary mouse anti-PLIN1 (1:500 dilution) overnight at 4 °C. The following day, sections were washed thrice with TBS containing 0.1% Triton X-100 for 10 min before incubation with goat anti-rat IgG conjugated to biotin (1:400 dilution) for 2 h at room temperature. Sections were then washed with TBS containing 0.1% Triton X-100 and incubated with goat anti-rabbit IgG conjugated with Alexa Flour 488 (1:400 dilution) and high-sensitivity streptavidin–HRP (1:500) for 2 h at room temperature. Sections were washed thrice with TBS containing 0.1% Triton X-100 for 10 min before being mounted with VECTASHIELD antifade mounting medium containing DAPI. All images were taken using a Zeiss LSM880 confocal microscope with Airyscan.

### Lipolysis assay

Glycerol released in the cell culture medium was quantified using the luminescence Glycerol-Glo assay (Promega), as per the manufacturer’s protocol. In brief, differentiated immortalized brown adipocytes were serum-starved for 2 h, washed with DPBS and treated as indicated in Krebs ringer buffer supplemented with 10 μM fatty-acid-free BSA pH 7.0. Cells were incubated with agonist for 30 min. Following incubation, 50 μl of cell supernatant was combined with an equal volume of glycerol detection reagent, resulting in a luminescent signal proportional to the amount of glycerol present. Luminescence was measured using the PHERAstar microplate reader (BMG Labtech). The glycerol concentration of each sample was calculated using the luminescence of glycerol standards with known concentrations.

### Fatty acid extraction

Differentiated adipocytes were serum-starved for 2 h, washed with DPBS and treated as indicated in Krebs ringer buffer with or without 100 μM fatty-acid-free BSA pH 7.0. Cell supernatants were collected at the specified time points and transferred to prechilled glass vials alongside thawed cell-free buffer blanks. Lipids were extracted from 50 μl of supernatant using a 1:1:2 (v/v) ratio of methanol containing 0.01% w/v butylated hydroxytoluene, water and dichloromethane containing heptadecanoic acid (C17:0) as an internal standard. In parallel, a quantitative external calibration curve was prepared using the same extraction procedure from a mixture of commercial standards. The extraction mixture was carefully vortexed followed by phase separation by centrifugation at 11,000*g* for 15 min at 4 °C. The bottom lipid-containing phase was transferred to a fresh glass vial using a glass pipette and subsequently dried under a gentle nitrogen stream at room temperature.

### GC–MS

Nonesterified fatty acids were analyzed following derivatization to pentafluorobenzyl esters. Unlike derivatization to fatty acid methyl esters, this technique does not disrupt esterified fatty acids, eliminating the requirement for preanalytical separation of these species^58,59^. Dried samples were first resuspended in 50 μl of a 1:1 mixture of 1% (v/v) solution of *N*,*N*-diisopropylethylamine and a 1% (v/v) solution of pentaflurobenzylbromine, both dissolved in acetonitrile, and left at room temperature for 30 min whilst protected from light. The solution was dried under a nitrogen stream at room temperature and resuspended in 100 μl of GC–MS-grade isooctane, vortexed and transferred to glass vials for GC–MS analysis. Pooled quality control samples were also prepared to run at the start, middle and end of each batch alongside the external calibration curve.

Samples were analyzed using an Agilent 8890 GC connected to a 5977B MSD system with a G3393B CI Upgrade kit. First, 5 μl of sample was injected in splitless mode onto a 90-m FastFAME column (Agilent, G3903-63013) using ultrapure helium (BOC) at a constant pressure of 46 psi. The following nonlinear temperature gradient was used: 50 °C before ramping to 160 °C at 50 °C min^−1^, followed by ramping to 230 °C at 10 °C min^−1^ and holding for 30 min, before ramping to 250 °C at 1 °C min^−1^ and holding for 16 min. The MS detector operated in negative chemical ionization mode with ultrapure methane (N5.5, BOC) as the collision gas. Collision gas flow rates and electron energies were determined by an instrument autotune at the start of each analytical run. The transfer line was maintained at 250 °C, MS source was maintained at 280 °C and MS quad was maintained at 200 °C. Fatty acids were detected as deprotonated anions ([M − H]^−^) following electron capture dissociation in full scan mode between 150 and 400 *m*/*z*. The lower limits of quantification for those fatty acids that were statistically significantly increased were as follows: C16:1n-7, 373 pM; C18:1n-9/n-7, 359 pM; C16:0, 390 pM.

### MS data processing

Raw MS spectra were converted to CDF files using Agilent ChemStation and processed using El Maven (Elucidata). Common mammalian saturated, monounsaturated and polyunsaturated fatty acids of chain length C12–C24 were identified using a combination of observed *m*/*z* and retention time matching to an internal library of authentic lipid standards and commercial standards run as part of the same analytical batch. Peak areas for each fatty acid species were normalized to the peak area of the internal standard heptadecanoate acid (C17:0) and then baseline-subtracted against the appropriate buffer blank from the same analytical run. For absolute quantification, calibration lines were constructed by linear regression before conversion of normalized signal intensities to concentrations using either direct comparison (if the fatty acid species was contained within the quantitative standard) or the nearest structurally similar lipid (for example, for C18:1n-7, the calibration line for C18:1n-9 was used).

### Statistics

Statistical analysis was performed using GraphPad Prism 10 software. Values are given as the mean ± s.e.m. Differences between two groups were assessed by Mann–Whitney test. Differences among three or more groups were assessed by one-way or two-way analysis of variance (ANOVA) followed by Dunnett’s or Šidák’s post hoc tests, as appropriate. Differences were considered significant for *P* values < 0.05.

### Reporting summary

Further information on research design is available in the Nature Portfolio Reporting Summary linked to this article.

## Online content

Any methods, additional references, Nature Portfolio reporting summaries, source data, extended data, supplementary information, acknowledgements, peer review information; details of author contributions and competing interests; and statements of data and code availability are available at 10.1038/s41589-025-01982-5.

## Supplementary information

Supplementary Information Supplementary Video 1 legend.

Reporting Summary

Supplementary Video 1 Supplementary Video 1: The intracellular pool of FFA4 associated with LDs is activated upon lipolysis induction with isoproterenol. HILO imaging of Halo–mGα_o_ recruitment to FFA4–YFP expressed in differentiated immortalized brown adipocytes. Cells were stimulated with 10 μM isoproterenol. Frames were acquired every 30 s. Playback is at 5 fps.

## Source data

Source Data Fig. 1 Statistical source data.

Source Data Fig. 2 Unprocessed western blot gels.

Source Data Fig. 3 Statistical source data.

Source Data Fig. 4 Statistical source data.

Source Data Fig. 5 Statistical source data.

Source Data Fig. 6 Statistical source data.

Source Data Extended Data Fig. 1 Statistical source data.

Source Data Extended Data Fig. 2 Statistical source data.

Source Data Extended Data Fig. 3 Statistical source data.

Source Data Extended Data Fig. 4 Statistical source data.

Source Data Extended Data Fig. 6 Unprocessed western blot gels.

Source Data Extended Data Fig. 7 Statistical source data.

Source Data Extended Data Fig. 8 Statistical source data.

Source Data Extended Data Fig. 9 Statistical source data.

## Data Availability

All imaging data supporting the figures in this study are available from the University of Birmingham Institutional Research Archive (UBIRA). Source data are provided with this paper.

## Code availability

MATLAB code specific to this study is available from GitHub (https://github.com/CalebiroLab/FFA4_intracrine/).
