# Supplementary material for: Intracrine FFA4 signaling controls lipolysis at lipid droplets
Source: Nat Chem Biol. 2025 Aug 5;22(1):109–19. doi: 10.1038/s41589-025-01982-5 (PMC12727528; doi:10.1038/s41589-025-01982-5)
Supplement: Supplementary file 1 — Supplementary Video 1 legend. [file 41589_2025_1982_MOESM1_ESM.pdf]

nature chemical biology

Supplementary information

https://doi.org/10.1038/s41589-025-01982-5

Intracrine FFA4 signaling controls lipolysis at lipid droplets

In the format provided by the authors and unedited

SUPPLEMENTARY INFORMATION

Supplementary Video 1: The intracellular pool of FFA4 associated with LDs is activated upon lipolysis induction with isoproterenol. HILO imaging of Halo-mGα_o recruitment to FFA4-YFP expressed in differentiated immortalized brown adipocytes. Cells were stimulated with 10 μM isoproterenol. Frames were acquired every 30 s. Playback, 5 frames/s.
