# Supplementary material for: Intracrine FFA4 signaling controls lipolysis at lipid droplets
Source: Nat Chem Biol. 2025 Aug 5;22(1):109–19. doi: 10.1038/s41589-025-01982-5 (PMC12727528; doi:10.1038/s41589-025-01982-5)
Supplement: Supplementary file 2 — Reporting Summary [file 41589_2025_1982_MOESM2_ESM.pdf]

nature portfolio

Corresponding author(s): Davide Calebiro, Shannon O'Brien

Last updated by author(s): Jun 25, 2025

Reporting Summary

Nature Portfolio wishes to improve the reproducibility of the work that we publish. This form provides structure for consistency and transparency in reporting. For further information on Nature Portfolio policies, see our Editorial Policies and the Editorial Policy Checklist.

Statistics

For all statistical analyses, confirm that the following items are present in the figure legend, table legend, main text, or Methods section.

n/a	Confirmed	
☐	☒	The exact sample size (n) for each experimental group/condition, given as a discrete number and unit of measurement
☐	☒	A statement on whether measurements were taken from distinct samples or whether the same sample was measured repeatedly
☐	☒	The statistical test(s) used AND whether they are one- or two-sided<br>*Only common tests should be described solely by name; describe more complex techniques in the Methods section.*
☒	☐	A description of all covariates tested
☐	☒	A description of any assumptions or corrections, such as tests of normality and adjustment for multiple comparisons
☐	☒	A full description of the statistical parameters including central tendency (e.g. means) or other basic estimates (e.g. regression coefficient) AND variation (e.g. standard deviation) or associated estimates of uncertainty (e.g. confidence intervals)
☐	☒	For null hypothesis testing, the test statistic (e.g. F, t, r) with confidence intervals, effect sizes, degrees of freedom and P value noted<br>*Give P values as exact values whenever suitable.*
☒	☐	For Bayesian analysis, information on the choice of priors and Markov chain Monte Carlo settings
☒	☐	For hierarchical and complex designs, identification of the appropriate level for tests and full reporting of outcomes
☒	☐	Estimates of effect sizes (e.g. Cohen's d, Pearson's r), indicating how they were calculated

Our web collection on statistics for biologists contains articles on many of the points above.

Software and code

Policy information about availability of computer code

Data collection	HILO images were acquired with MetaMorph 7.10.2.240. SIM Images were acquired with NIS-Elements (Nikon) and reconstructed with ARIVIS (Zeiss). Confocal images were acquired with a laser scanning confocal Zeiss LSM880 Confocal Microscope with Airyscan. Western blots were imaged on an Odyssey imaging system. BRET and luminescent readings data was acquired with Pherastar FSX Mars data analysis 3.32 (BMG); EbBRET data was acquired with a Spark multimode microplate reader (Tecan). Intracellular versus plasma membrane FFA4 was quantified using Ilastik 1.4.0 and subsequently analysed using Metamorph 7.10.5.476
Data analysis	Statistical analyses were performed with GraphPad Prism 10.4.0. Microscopy images were analyzed using ImageJ 1.54F

For manuscripts utilizing custom algorithms or software that are central to the research but not yet described in published literature, software must be made available to editors and reviewers. We strongly encourage code deposition in a community repository (e.g. GitHub). See the Nature Portfolio guidelines for submitting code & software for further information.

Data

Policy information about availability of data

All manuscripts must include a data availability statement. This statement should provide the following information, where applicable:

- Accession codes, unique identifiers, or web links for publicly available datasets
- A description of any restrictions on data availability
- For clinical datasets or third party data, please ensure that the statement adheres to our policy

All data presented in graphs within the Figures and Extended Data Figures are available in the source data files. All blots and gels presented throughout are supplied as un-cropped versions in the Source data files. Additionally, all image data used to classify subcellular localization of FFA4 and/or mini-G translocation using ilastik classification and subsequent MATLAB quanitifications, have been deposited on to the open access University of Birmingham Research Archive (UBIRA) eData Repository. MATLAB code used has been deposited to GitHub

Human research participants

Policy information about studies involving human research participants and Sex and Gender in Research.

Reporting on sex and gender	No sex- and gender-based analyses have been performed
Population characteristics	The study does not involve research on human populations
Recruitment	The study does not involve research on human populations
Ethics oversight	The study does not involve research on animals or humans

Note that full information on the approval of the study protocol must also be provided in the manuscript.

Field-specific reporting

Please select the one below that is the best fit for your research. If you are not sure, read the appropriate sections before making your selection.

☒ Life sciences ☐ Behavioural & social sciences ☐ Ecological, evolutionary & environmental sciences

For a reference copy of the document with all sections, see nature.com/documents/nr-reporting-summary-flat.pdf

Life sciences study design

All studies must disclose on these points even when the disclosure is negative.

Sample size	For animal studies, no statistical methods were used to predetermine sample size. Animal numbers were chosen based on previously published research. For in vivo and in vitro experiments, at least three distinct samples were used. For all cell line experiments, three or more independent biological experiments were performed.
Data exclusions	No samples or data were excluded form this study
Replication	All findings were replicated as specified in the figure legends. When representative data are shown, the number of times that the experiment was repeated with similar results is stated in the figure legend.
Randomization	For single-cell imaging experiments cells were chosen randomly before assignment to a given experimental group. For the other experiments performed in this study randomization is usually not necessary.
Blinding	BRET and imaging data were analysed using standardized and automated software pipelines with limited or no user intervention, respectively, to minimise bias.

Reporting for specific materials, systems and methods

We require information from authors about some types of materials, experimental systems and methods used in many studies. Here, indicate whether each material, system or method listed is relevant to your study. If you are not sure if a list item applies to your research, read the appropriate section before selecting a response.

Materials & experimental systems

n/a	Involved in the study
☐	☒ Antibodies
☐	☒ Eukaryotic cell lines
☒	☐ Palaeontology and archaeology
☐	☒ Animals and other organisms
☒	☐ Clinical data
☒	☐ Dual use research of concern

Methods

n/a	Involved in the study
☒	☐ ChIP-seq
☒	☐ Flow cytometry
☒	☐ MRI-based neuroimaging

Antibodies

Antibodies used

UCP1 Polyclonal Antibody (Cat # PA1-24894), Perilipin-1 Monoclonal Antibody (Cat # MA5-27861, Clone GT2781), Goat anti-Rabbit Secondary Antibody Alexa Fluor 488 (Cat # A-11008), Goat anti-Rat Secondary Antibody Biotin (Cat # 31830), Pierce High Sensitivity Streptavidin-HRP (Cat # 21130) were from Thermo Fisher Scientific Ltd. IRDye Donkey anti-rabbit secondary antibody (Cat # 926-32213) and Donkey anti-rat secondary antibody (Cat # 926-32219) were from LI-COR Biotechnology; rabbit anti-Na+/K+ ATPase antibody (ab76020) was from Abcam; HA monoclonal antibody (Cat # 11867423001, clone 3F10) was from Roche.

Validation

The HA antibodies against FFA4-HA and FFA2-HA were validated using FFA2/FFA4 knockout control animals. Secondary antibodies were validated through experiments using secondary antibodies alone without primary antibodies. Additionally, specificity was confirmed by using multiple antibodies against target proteins (e.g., UCP1, NA+/K+ ATPase and PLIN1), all yielding consistent detection and staining patterns.

All antibodies have been validated by the manufacturers and detailed information can be found on the website from manufactures listed below:
UCP1 Polyclonal Antibody: https://www.thermofisher.com/antibody/product/UCP1-Antibody-Polyclonal/PA1-24894
Perilipin-1 Monoclonal Antibody: https://www.thermofisher.com/antibody/product/Perilipin-1-Antibody-clone-GT2781-Monoclonal/MA5-27861
Goat anti-Rabbit Secondary Antibody Alexa Fluor 488: https://www.thermofisher.com/antibody/product/Goat-anti-Rabbit-IgG-H-L-Cross-Adsorbed-Secondary-Antibody-Polyclonal/A-11008
Goat anti-Rat Secondary Antibody Biotin: https://www.thermofisher.com/antibody/product/Goat-anti-Rabbit-IgG-H-L-Cross-Adsorbed-Secondary-Antibody-Polyclonal/31830)
Pierce High Sensitivity Streptavidin-HRP: https://www.thermofisher.com/order/catalog/product/21130
IRDye Donkey anti-rabbit secondary antibody: https://shop.licor.com/bio/reagents/irdye-800cw-donkey-anti-rabbit-igg-secondary-antibody
IRDye Donkey anti-rat secondary antibody: https://shop.licor.com/bio/reagents/irdye-800cw-goat-anti-rat-igg-secondary-antibody
Rabbit anti-Na+/K+ ATPase antibody: https://www.abcam.com/en-us/products/primary-antibodies/sodium-potassium-atpase-antibody-ep1845y-plasma-membrane-loading-control-ab76020
HA monoclonal antibody: https://www.sigmaaldrich.com/GB/en/product/roche/roahaha?srsltid=AfmBOoqqjCKs_Nkq4T8K53UZNUFcNix_jeNjGYSfMbGhjbzaNNKaK3f5

Eukaryotic cell lines

Policy information about cell lines and Sex and Gender in Research

Cell line source(s)

HEK293T and 3T3-L1 were from ATCC. Immortalized mouse brown preadipocytes were obtained from Patrick Seal. HEK293SL are a subclone derived from regular HEK293 cells (Ad5 transformed) and were obtained from Michel Bouvier.

Authentication

Cells were cultured for a maximum of 25 passages. No authentication was used.

Mycoplasma contamination

Cells were routinely tested for mycoplasma contamination by commercially available testing kits, and verified as mycoplasma negative.

Commonly misidentified lines
(See ICLAC register)

The cell lines used in this study are not listed in the the database of commonly misidentified cell lines.

Animals and other research organisms

Policy information about studies involving animals; ARRIVE guidelines recommended for reporting animal research, and Sex and Gender in Research

Laboratory animals

Adult C57BL/6 mice (12-16 weeks)

Wild animals

This study did not involve any wild animals

Reporting on sex

Both male and female mice were used in this study. Samples were collected from adult mice (12-16 weeks old). No sex based differences were found in the measured parameters. Total number of mice are 51 (females: 21, males: 30).

Field-collected samples	The study did not involve samples collected from the field.
Ethics oversight	Breeding, maintenance, and killing of mice conformed to the United Kingdom Home Office regulations (Project license number: PP0894775).

Note that full information on the approval of the study protocol must also be provided in the manuscript.
