# Supplementary material for: Intracrine FFA4 signaling controls lipolysis at lipid droplets
Source: Nat Chem Biol. 2025 Aug 5;22(1):109–19. doi: 10.1038/s41589-025-01982-5 (PMC12727528; doi:10.1038/s41589-025-01982-5)
Supplement: Supplementary file 14 — Unprocessed western blot gels. [file 41589_2025_1982_MOESM14_ESM.pdf]

Extended data Figure 6a

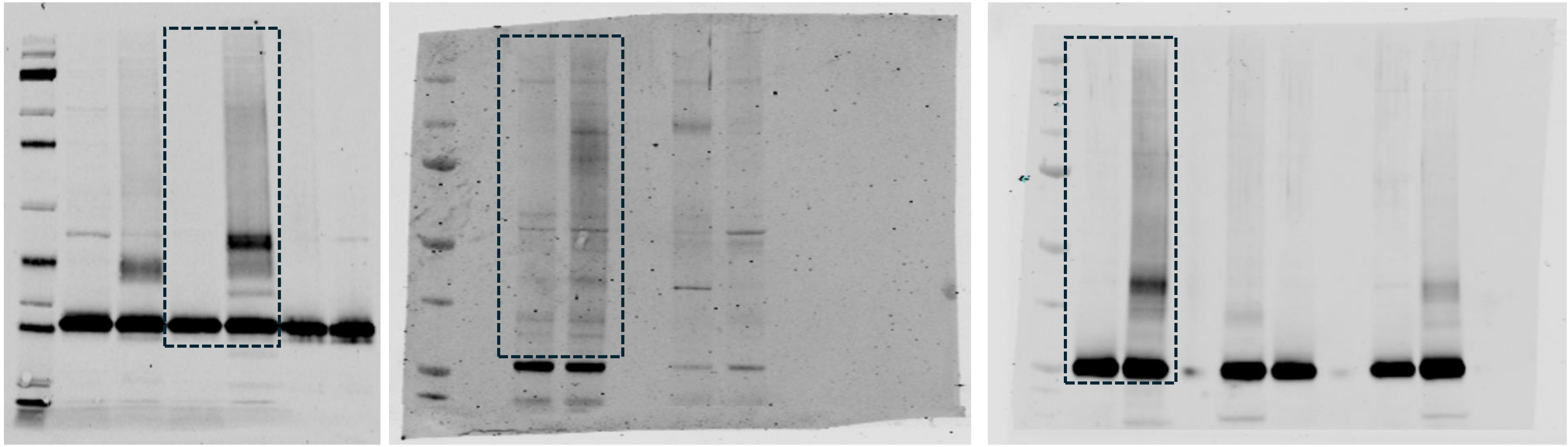

HA monoclonal antibody (Roche, Cat # 11867423001)

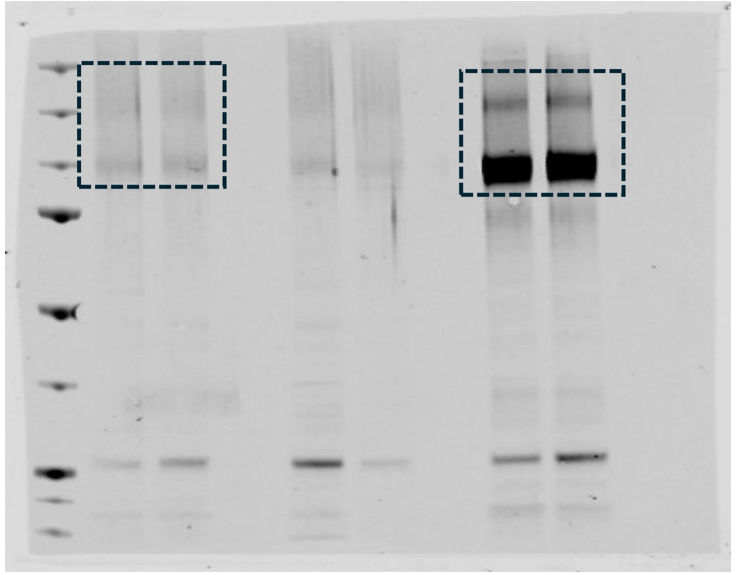

Rabbit anti-Na^{+}/K^{+} ATPase antibody (ab76020, Abcam)

Extended data Figure 6b

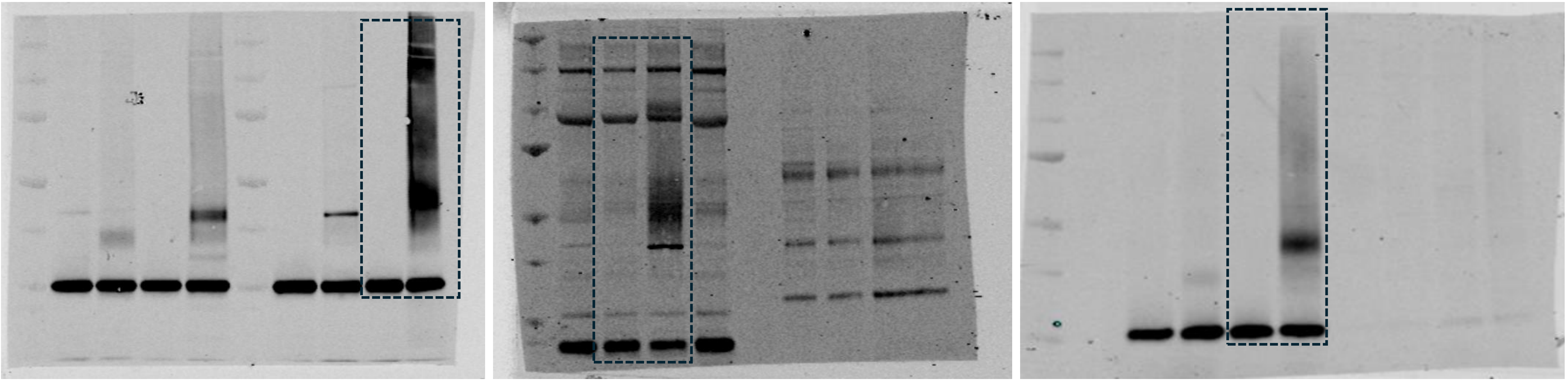

HA monoclonal antibody (Roche, Cat # 11867423001)

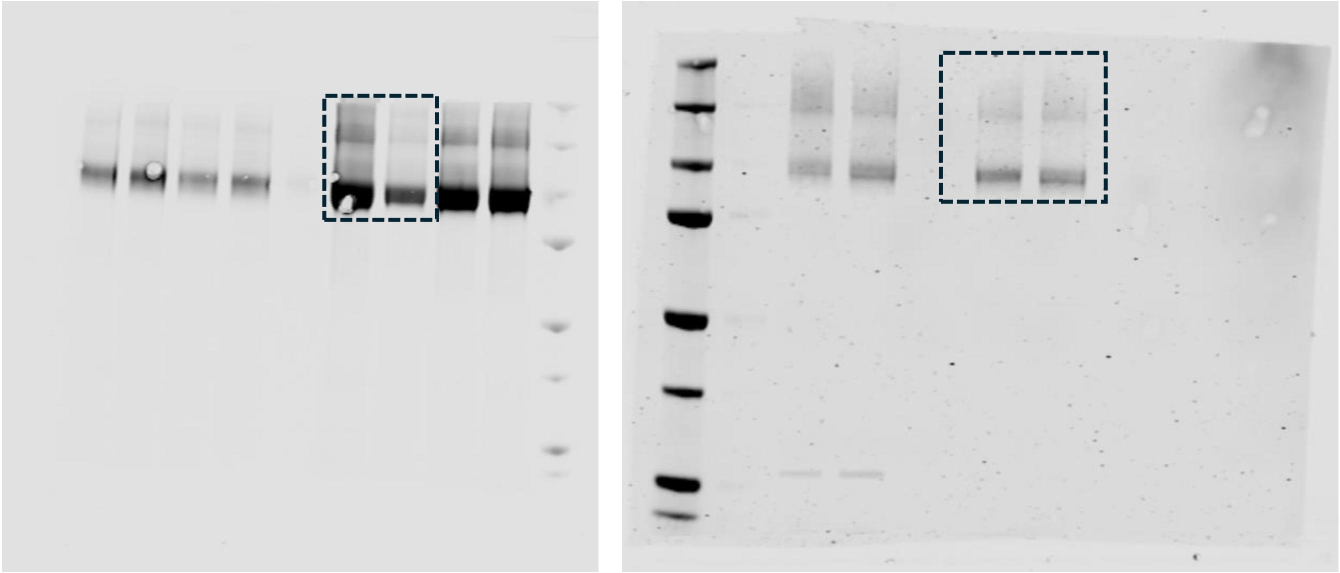

Rabbit anti-Na^+/K^+ ATPase antibody (ab76020, Abcam)
